# Bevacizumab in Combination with Modified FOLFOX6 in Heavily Pretreated Patients with HER2/Neu-Negative Metastatic Breast Cancer: A Phase II Clinical Trial

**DOI:** 10.1371/journal.pone.0133133

**Published:** 2015-07-17

**Authors:** Ting Li, Biyun Wang, Zhonghua Wang, Joseph Ragaz, Jian Zhang, Si Sun, Jun Cao, Fangfang Lv, Leiping Wang, Sheng Zhang, Chen Ni, Zhenhua Wu, Jie Xie, Xichun Hu

**Affiliations:** 1 Department of Medical Oncology, Fudan University Shanghai Cancer Center, Shanghai, 200032, China; 2 Department of Oncology, Shanghai Medical College, Fudan University, Shanghai, 200032, China; 3 Faculty of Medicine, School of Population & Public Health, University of British Columbia, Vancouver, B.C., V6T 1Z4, Canada; University Campus Bio-Medico, ITALY

## Abstract

**Background:**

Bevacizumab combined with modified FOLFOX6 is a standard regimen for colorectal cancer. The present study was to determine the efficacy and safety of bevacizumab-modified FOLFOX6 regimen in heavily pretreated patients with human epidermal growth factor receptor 2 (HER2/neu)-negative MBC.

**Methods:**

Bevacizumab, 5 mg/kg every two weeks or 7.5 mg/kg every three weeks, was administered with modified FOLFOX6 (oxaliplatin 85 mg/m^2^, leucovorin 400 mg/m^2^, 5-FU 400 mg/m^2^ on day 1, followed by 5-FU 2400 mg/m^2^ intravenous infusion over 46 hours every 2 weeks) to patients who failed at least 1 chemotherapy regimen in the metastatic setting. The primary objective was progression free survival (PFS). Secondary objectives included objective response rate (ORR), clinical benefit rate (CBR), overall survival (OS), safety, and the change of tumor size and Eastern Cooperative Oncology Group (ECOG) performance status.

**Results:**

69 patients were enrolled. The median PFS was 6.8 months (95% CI, 5.0 to 8.5 months), ORR was 50.0% and median OS was 10.5 months (95% CI, 7.9 to 13.1 months). Patients showing objective responses had a 4.2-month median PFS gain and 5.7-month median OS gain compared with those who did not (*P* < 0.05). Grade 3 or 4 adverse events occurring in more than one patient were neutropenia (53/69, 76.8%), leukopenia (36/69, 52.2%), thrombocytopenia (13/69, 18.8%), anemia (3/69, 4.3%) and hypertension (3/69, 4.3%).

**Conclusions:**

Adding bevacizumab to modified FOLFOX6 does have significant anti-tumor activity and good safety profile in heavily pretreated HER2/neu-negative MBC patients. Further trials are required to confirm whether the high ORR can translate into a long-term PFS and even OS benefit.

**Trial Registration:**

www.clinicaltrials.gov NCT01658033

## Introduction

A majority of metastatic breast cancer (MBC) patients will succumb to their disease within 2 years of diagnosis [1]. Despite significant efficacy of taxanes and anthracyclines, nearly all patients will eventually develop drug resistance, and subsequent chemotherapy regimens are frequently required. Oxaliplatin, 5-fluorouracil (5-FU) and leucovorin (LV) comprise a series of FOLFOX regimens for adjuvant or palliative treatment in colorectal cancer, with high efficacy and good safety profile. Data showed that those agents were well tolerated and potentially active in heavily pretreated MBC [2–4]. A phase II clinical trial in our institution demonstrated that modified FOLFOX6 (mFOLFOX6) served as a potentially effective salvage regimen with favorable toxicity in heavily pretreated MBC patients [5].

Bevacizumab, a humanized monoclonal antibody, produces angiogenesis inhibition by inhibiting vascular endothelial growth factor A (VEGF-A) [6]. Adding bevacizumab to the FOLFOX4 and mFOLFOX6 regimens are shown to be more effective for patients with metastatic colorectal cancer than FOLFOX4 and mFOLFOX6 regimens [7–9]. However, its long-term impact in breast cancer is still not clear. In the neoadjuvant setting, adding bevacizumab to chemotherapy significantly increases the pathological complete response rate in human epidermal growth factor receptor 2 (HER2/neu)-negative breast cancer [10–12]. In metastatic setting, bevacizumab combined with weekly paclitaxel for stage IV disease has a median progression free survival (PFS) of 10.4 to 11.8 months [13–15], which is listed as one of the first-line treatments by National Comprehensive Cancer Network (NCCN) guideline [16]. Although none of all published bevacizumab-based trials shows prolongation of overall survival (OS), its value in control of disease has been consistently confirmed whether combined with different chemotherapeutic agents or used in different clinical settings, like first- and second-line [17–19], and even later setting [20]. Further, a lot of studies are actively ongoing to explore bevacizumab maintenance therapy and drug resistance [21–23], other anti-angiogenesis agents, and relevant predictive biomarkers [24, 25].

Given the above encouraging data of bevacizumab and a series of FOLFOX regimens, the present phase II study was initiated to evaluate the efficacy and safety of combining bevacizumab with mFOLFOX6 (bevacizumab-mFOLFOX6) for patients with HER2/neu-negative MBC who had received one to six cytotoxic regimens in metastatic setting.

## Patients and Methods

### Patients

Inclusion criteria included patients with a histologically confirmed HER2/neu-negative MBC, age ≥ 18 years, more than 12-week of life expectancy, Eastern Cooperative Oncology Group (ECOG) performance status of 0, 1 or 2 [26], and at least one extracranial measurable disease according to the Response Evaluation Criteria in Solid Tumors (RECIST) version 1.1 [27], that had not been previously irradiated. Enrolled patients had to have at least 1 prior chemotherapy regimens for the metastatic disease, and were pretreated with anthracyclines and taxanes. Patients were required to complete all prior chemotherapy or radiotherapy at least 3 weeks before study entry.

Patients were excluded if they had no objective response to the prior treatments of oxaliplatin, capecitabine or continuous infusion of 5-FU. Patients, whose cumulative doses of doxorubicin and epirubicin exceeded 360 mg/m^2^ and 720 mg/m^2^ respectively, were also excluded.

### Study Design and Objectives

This was an open-label, single-arm, phase II study (Trial Registration: http://www.clinicaltrials.gov NCT 01658033). The recruitment was from July 6, 2012 to November 29, 2013. Patients were treated with bevacizumab, 5 mg/kg every two weeks or 7.5 mg/kg every three weeks on day 1, and mFOLFOX6 every 2 weeks. It included oxaliplatin 85 mg/m^2^, LV 400 mg/m^2^, 5-Fu 400 mg/m^2^ intravenous bolus on day 1, followed by 5-Fu 2400 mg/m^2^ intravenous infusion over 46 hours. The bevacizumab-mFOLFOX6 regimen was repeated until progression disease (PD), unacceptable toxicity, death, or withdrawal of informed consent. Overall, two dose reductions for toxicities were allowed; if dose reduction was required for the third time, the treatment was discontinued. The primary objective was PFS. Secondary objectives included ORR, clinical benefit rate (CBR), OS, toxicity profiles, the change of tumor size and ECOG performance status from baseline. The follow-up time was from the date of first patient enrollment to March 10, 2015. This study was performed in accordance with the International Conference on Harmonization Good Clinical Practice guidelines, the Declaration of Helsinki (1996 version), applicable local regulatory requirements and laws. The study was approved by Fudan University Shanghai Cancer Center Ethic Committee for Clinical Investigation on July 2, 2014 and was carried out in Fudan University Shanghai Cancer Center. In the July, we initiated this trial and registered this trial on the web. There was no delay in the registration of this trial. Written informed consents were obtained from all patients prior to enrollment.

### Assessments and Data Collection

Patients underwent clinical examination and radiographic assessment of measurable disease for tumor response every 2 cycles or clinically indicated. The cycle was counted by the treatment of mFOLFOX6 regimen and duration of one cycle was 28 days with twice medications of mFOLFOX6. PFS was defined as the time from enrollment to the first documented date of disease progression or death from any cause. OS was defined as the time from enrollment to the date of death from any cause. ORR was defined as the percentage of patients who achieved complete response (CR) and partial response (PR) by RECIST, version 1.1 [27]. CBR was defined as the percentage of patients who achieved CR, PR and stable disease (SD) ≥ 24 weeks by RECIST, version 1.1 [27]. The change of tumor size was defined as the change of the sum of longest dimensions for target lesions from baseline to maximal tumor shrinkage. Adverse events (AEs) were evaluated, graded and recorded according to National Cancer Institute Common Terminology Criteria for Adverse Events (CTCAE), version 4.03 [28]. The ECOG performance status was recorded at baseline and before every treatment cycle [26].

### Statistics

Compared to the pilot mFOLFOX6 regimen without bevacizumab [5], the planned sample size of 62 patients treated with new bevacizumab-mFOLFOX6 regimen would allow detecting an increase of 2 months in median PFS, with a 5% significance level at 95% power and 10% patient dropout rate. PFS, OS and safety end points were analyzed in patients who received at least one dose of treatment. ORR and CBR were analyzed in patients who had at least one response evaluation data. Statistical analysis of 2×2 contingency tables of categorical variables was carried out using Fisher’s exact test. Median PFS, median OS and their 95% confidence intervals were calculated using Kaplan–Meier method. PFS and OS differences were calculated using Kaplan–Meier method with log-rank test. Factors with *P*<0.1 in the univariate analysis were examined with Cox proportional hazard model which defined independent predictive factors. The change of ECOG performance status after chemotherapy was analyzed by Wilcoxon signed-rank test. All statistical tests were 2-tailed, with significance defined as *P <* 0.05. The Statistical Package for the Social Sciences software (SPSS) version 19.0 was used for all statistical analyses [29].

## Results

### Characteristics of Patients at Baseline

**Fig 1:**
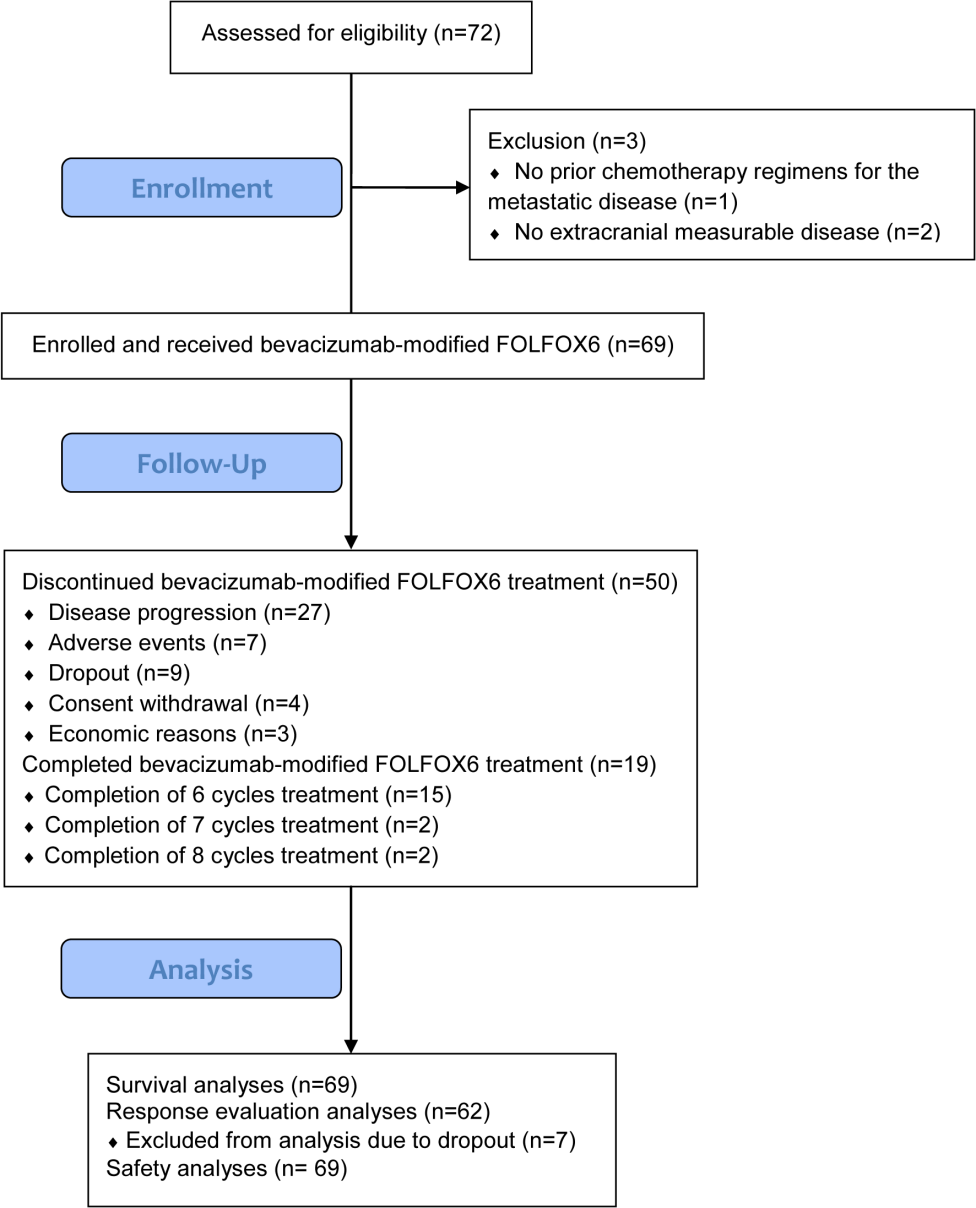
The flow diagram of the present phase II clinical study.

From July 2012 to November 2013, 72 heavily pretreated patients with HER2/neu-negative MBC were enrolled. Three patients were excluded from analysis because one had no prior chemotherapy regimens for the metastatic disease and the other two had no extracranial measurable disease according to the RECIST version 1.1 (Fig 1). The cutoff date for analysis was March 10, 2015, resulting in a median follow-up time of 10.5 months. The characteristics of patients at baseline are listed in Table 1.

**Table 1:** Patient Characteristics at baseline (*N* = 69). Abbreviations: ECOG, Eastern Cooperative Oncology Group; MBC, metastatic breast cancer.

Characteristics	*No*.	%
Age (Median, range)	49, 28–73	
< 60 years	56	81.2
≥ 60 years	13	18.8
Menstruation status		
Post-menopausal	43	62.3
Pre-menopausal	26	37.7
ECOG performance status		
0	2	2.9
1	61	88.4
2	6	8.7
Molecular subtype		
Luminal A	5	7.2
Luminal B	32	46.4
Triple-negative	32	46.4
Number of metastatic sites		
< 3	25	36.2
≥ 3	44	63.8
Metastatic sites		
Lymph nodes	49	71.0
Bone	42	60.9
Liver	40	58.0
Lung	36	52.3
Chest wall recurence	30	43.5
Pleura	16	23.2
Brain	7	10.1
Contralateral breast	3	4.3
Others	2	2.9
Visceral metastasis		
Yes	58	84.1
No	11	15.9
Disease-free interval*		
> 12 months	47	74.6
≤ 12 months	16	25.4
Prior Adjuvant Chemotherapy		
Anthracyclines	62	89.9
Taxanes	44	63.8
Prior chemotherapy regimens for MBC (Median, range)	2, 1–6	
≥ 3	31	44.9
2	17	24.6
1	21	30.4
Prior chemotherapy drug		
Taxanes	68	98.6
Anthracyclines	65	94.2
Gemcitabine	48	69.6
Capecitabine	32	46.4
Vinorelbine	31	44.9

* There were no disease-free interval data for 6 patients, because they had metastatic sites when first diagnosed and did not receive radical surgery.

### Treatment Exposure

69 patients received at least one dose of assigned medical treatment. At the last follow-up on March 10, 2015, patients received a median of 4.0 treatment cycles (range, 0.5 to 8.0). Seven cases had their treatment discontinued due to AEs, including 4 cases with grade 3 or 4 thrombocytopenia who had not recovered within additional two weeks of delays; these 4 cases received 1.0, 2.0, 4.5 and 5.0 cycles of bevazicumab-mFOLFOX6 regimen, respectively; 2 cases with cardiac events (1 with 18% decrease in left ventricular ejection fraction compared with baseline; 1 with moderate supraventricular ectopic beats with ST segment depression and T wave changes); these 2 patients had 0.5 and 2.0 treatment cycles before therapy was discontinued; 1 case with grade 3 wound healing complications and the patient received 2 treatment cycles before therapy was discontinued. The relative dose intensity was 99.9% for bevacizumab (range, 81.6% to 110.1%), 99.1% for 5-FU (range, 82.6% to 104.82%), 99.1% for LV (range, 87.6% to 103.8%), and 98.1% for oxaliplatin (range, 71.8% to 105.0%), respectively.

### Efficacy

**Fig 2:**
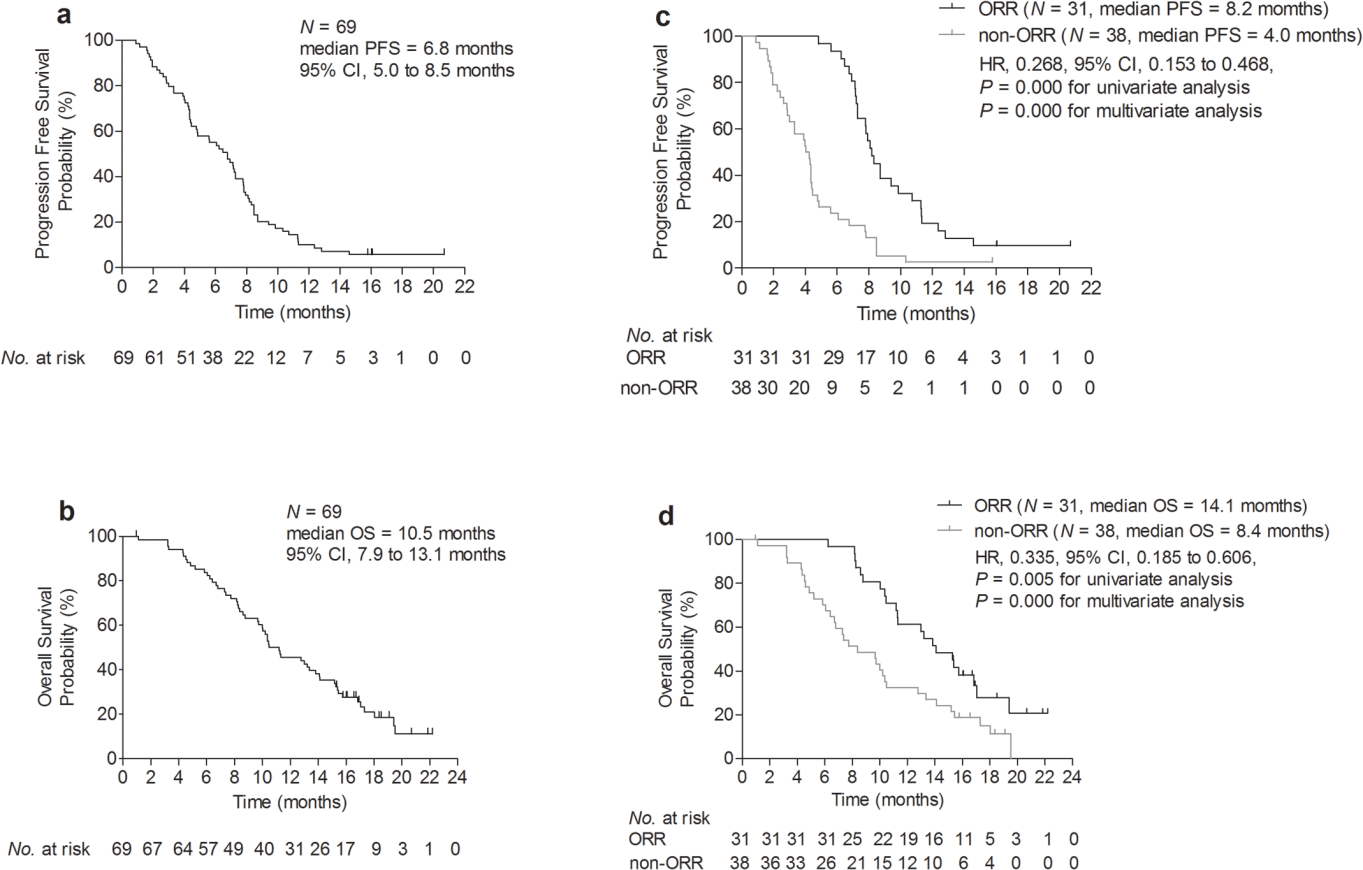
Kaplan–Meier plot of PFS (a) and OS (b) for all patients and Kaplan-Meier plot of PFS (c) and OS (d) in patients with ORR versus non-ORR.

**Fig 3:**
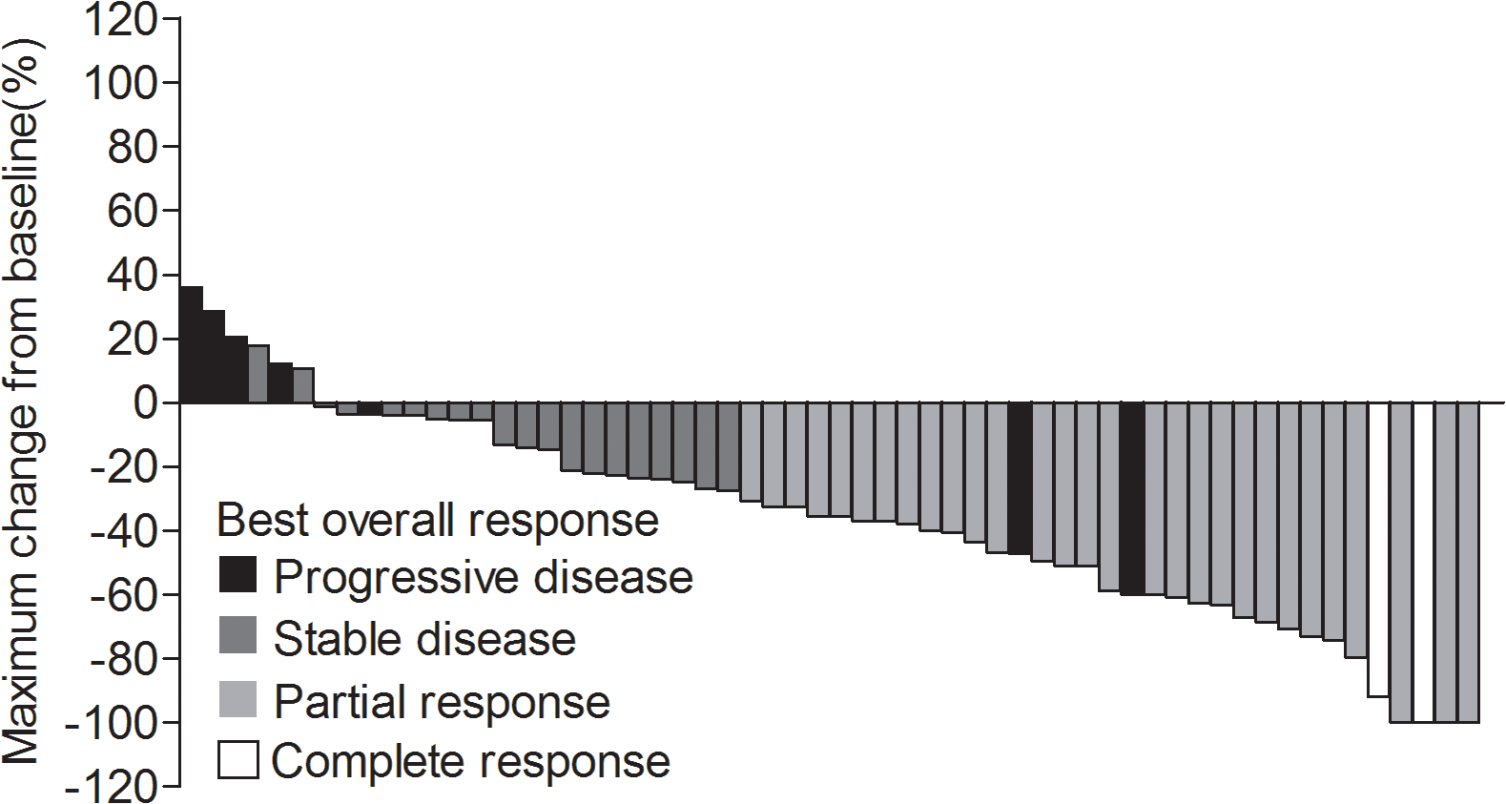
Waterfall plot of the maximum change in tumor size showed best overall responses in each patient.

Of the 69 enrolled patients, 94.2% patients (65/69) had had PD and 79.7% patients (55/69) had died at data cutoff. As shown in Fig 2A and 2B, the median PFS was 6.8 months (95% confidence interval [CI], 5.0 to 8.5 months) and the median OS was 10.5 months (95% CI, 7.9 to 13.1 months). Fig 2C and 2D showed that patients showing objective responses had a longer PFS and OS than those who did not (*P* < 0.05). Among 62 patients eligible for response evaluation, an ORR was achieved in 31 (50.0%) patients including 2 complete response and 29 partial responses. The CBR was achieved in 35 (56.5%) patients including 2 CR, 29 PR and 4 SD ≥24 weeks. The change in tumor size (sum of longest dimensions for target lesions) from baseline to maximal tumor shrinkage was shown in Fig 3.

### Factors predicting clinical outcome

According to the clinical significance and literature published [24, 30, 31], age, ECOG performance status, disease free interval, molecular subtype, menopausal status, metastatic sites at baseline (visceral metastases, yes vs. no), number of metastasis sites (< 3 vs. ≥ 3), objective response status (ORR vs. non-ORR), lines of bevacizumab-mFOLFOX6 regimen treatment (2 vs. ≥ 3), prior chemotherapy drug, hypertension and proteinuria were included for analysis (S1 Table and S2 Table). In univariate analysis, PFS and OS was significantly longer among patients achieving objective response (for PFS, ORR vs. non-ORR, 8.2 month vs. 4.0 month, *P* = 0.000; for OS, ORR vs. non-ORR, 14.1 month vs. 8.4 month, *P* = 0.005) [Fig 2C and 2D]. In multivariable analysis, independent factors for PFS with statistical significance were objective response (HR, 0.268, 95% CI: 0.153 to 0.468, *P* = 0.000), non-TNBC pathology (HR, 0.484, 95% CI: 0.281 to 0.833, *P* = 0.009) and number of metastasis sites (HR, 1.210, 95% CI: 1.004 to 1.459, *P* = 0.045). Independent factors for OS with statistical significance were objective response (HR, 0.335, 95% CI: 0.185 to 0.606, *P* = 0.000), older age (HR, 0.954, 95% CI: 0.926 to 0.983, *P* = 0.002) and number of metastasis sites (HR, 1.420, 95% CI: 1.156 to 1.743, *P* = 0.001).

### Safety

AEs are presented in Table 2. Most AEs were grade 1 or 2. Grade 3 or 4 AEs occurring in more than one patient were neutropenia (53/69, 76.8%), leukopenia (36/69, 52.2%), thrombocytopenia (13/69, 18.8%), anemia (3/69, 4.3%), hypertension (3/69, 4.3%) and febrile neutropenia (2/69, 2.9%). There were no severe AEs due to bevacizumab-mFOLFOX6 regimen, and specifically no severe AEs related patient deaths, neither during treatment nor within 1 month after treatment. The common AEs, which were reported previously and may be related to bevacizumab [8, 32–35], were mostly mild (grade 1 or 2) and included bleeding (23/69, 33.3%), cardiac events (22/69, 31.9%) and proteinuria (11/69, 15.9%). The bleeding was primarily limited to minor mucosal oozing that did not require medical intervention. Cardiac events and proteinuria were limited to clinical documented laboratory reports with no symptoms. There were only one case of grade 3 wound healing complications and three cases of grade 3 hypertension. Furthermore, the ECOG performance status had not increased substantially over cycles of chemotherapy (*P* = 0.414).

**Table 2:** AEs (*N* = 69). Note: This table included AEs occurring between the date of the first dose and 30 days following the last dose of study treatment. Abbreviations: AEs, adverse events; NA, not applicable.

AEs	Grade					
	1 or 2		3 or 4		Total	
	*No*.	%	*No*.	%	*No*.	%
Non-hematologic						
Nausea	30	43.5	0	0.0	30	43.5
Sensory neuropathy	28	40.6	0	0.0	28	40.6
Vomiting	25	36.2	0	0.0	25	36.2
Bleeding	23	33.3	0	0.0	23	33.3
Cardiac events	22	31.9	0	0.0	22	31.9
Fatigue	19	27.5	1	1.4	20	28.9
Diarrhea	18	26.1	0	0.0	18	26.1
Mucositis	10	14.5	1	1.4	11	15.9
Proteinuria	11	15.9	0	0.0	11	15.9
Abdominal pain	10	14.5	0	0.0	10	14.5
Liver dysfunction	9	13.0	0	0.0	9	13.0
Hypertension	5	7.2	3	4.3	8	11.6
Fever	8	11.6	0	0.0	8	11.6
Cough	5	7.2	0	0.0	5	7.2
Alopecia	5	7.2	NA	NA	5	7.2
Anorexia	4	5.8	0	0.0	4	5.8
Rash	4	5.8	0	0.0	4	5.8
Hand-foot syndrome	3	4.3	0	0.0	3	4.3
Myalgia	3	4.3	0	0.0	3	4.3
Vertigo	3	4.3	0	0.0	3	4.3
Infection	3	4.3	0	0.0	3	4.3
Arthralgia/Bone pain	2	2.9	0	0.0	2	2.9
Febrile neutropenia	NA	NA	2	2.9	2	2.9
Constipation	2	2.9	0	0.0	2	2.9
Headache	1	1.4	0	0.0	1	1.4
Abdominal distension	1	1.4	0	0.0	1	1.4
Hoarseness	1	1.4	0	0.0	1	1.4
Anaphylaxis	NA	NA	1	1.4	1	1.4
Nail discoloration	1	1.4	NA	NA	1	1.4
Tinnitus	1	1.4	0	0.0	1	1.4
Allergic reaction	1	1.4	0	0.0	1	1.4
Palpitations	1	1.4	NA	NA	1	1.4
Wound healing complications	0	0.0	1	1.4	1	1.4
Hematologic						
Leukopenia	29	42.0	36	52.2	65	94.2
Neutropenia	11	15.9	53	76.8	64	92.8
Thrombocytopenia	24	34.8	13	18.8	37	53.6
Anemia	31	44.9	3	4.3	34	49.3

## Discussion

This open label, single-arm, phase II study evaluated the efficacy and safety of bevacizumab-mFOLFOX6 regimen in patients with HER-2/neu-negative MBC. Although all patients were heavily pretreated, the bevacizumab-mFOLFOX6 regimen was tolerated reasonably well, with an ORR of 50.0% and CBR of 56.5%. The median PFS was 6.8 months, 1.8-month longer than the anticipated median PFS of 5-month, a result meeting our primary endpoint. Moreover, longer PFS and OS were found in patients with ORR, which was an independent predictor in multivariable analyses. When compared with our previously published study, adding bevacizumab to mFOLFOX6 regimen resulted in 32% improvement in ORR, 3.8-month improvement in median PFS [5].

Compared with other regimens for stage IV disease, our regimen produces comparable or possibly better results. Two trials restricted to second- or third-line treatment showed a median PFS of shorter than 5 months and an ORR of less than 30% [20, 36]. von Minckwitz et al reported an ORR of 21% and a median PFS was 6.3 month with bevacizumab-based regimen as second-line treatment [22], but our trials included a population with poorer prognosis (eg, patients with more than 84% having visceral disease, about 63% having ≥ 3 metastatic sites, 45% undergoing fourth-line therapy, 98% pretreated with taxane therapy and 94% with anthracyclines therapy). Eribulin, one standard drug in later setting, is still not commercially available in China. In terms of OS, bevacizumab-mFOLFOX6 regimen were comparable, but had stronger anti-tumor activity than single-agent eribulin (ORR, 14.1%; median PFS, 2.6 months; S3 Table) [37, 38]. With other chemotherapeutic agents including gemcitabine, vinorelbine, vinflunine and paclitaxel, patients in our trial showed a 2 to 4 months improvement in PFS, and 27% to 50% improvement in ORR (S3 Table) [39–45].

Continuous VEGF inhibition is necessary to maximize the benefit of bevacizumab [46], and recent studies provided evidences, showing that bevacizumab maintenance improved both PFS and OS [47]. In the context of no OS benefit with bevacizumab in other trials, it is risingly important to characterize validated biomarkers predicting bevacizumab treatment outcome. In our present study, the heavily-pretreated HER2/neu-negative breast cancer patients achieving objective responses had a 4.2-month median PFS gain (*P* = 0.000) and 5.7-month median OS gain (*P* = 0.005) when compared with those who did not achieve objective responses. Other clinicopathological factors favorably linked to longer PFS included non-TNBC pathology and number of metastasis sites in our cohort. Older age, objective response status and number of metastasis sites were predictive factors for longer OS. It was reported that TNBC patients with particularly prominent VEGFA amplification was associated with poor outcomes when treated with paclitaxel with or without bevacizumab [48]. Therefore, future larger studies were required to confirm that if these encouraging benefits of bevazcizumab-mFOLFOX6 regimen would translate into a long-term survival gains in patients with HER2/neu-negative MBC.

With regard to safety, AE profile of bevacizumab-mFOLFOX6 regimen did not change significantly compared to the previous data published for mFOLFOX6 alone [5] and the increase of grade 3–4 toxicities was restricted to neutropenia (S4 Table) [5]. When compared with the same bevacizumab-mFOLFOX6 regimen in colorectal cancer, our present study in MBC resulted in a somehow higher hematological toxicities (S5 Table) [9, 49–51]. However, febrile neutropenia and dose discontinuation rates were very low in our trial (S5 Table) [9, 49–51], and patients’ ECOG performance status did not worsen over cycles of bevazicumab-mFOLFOX6 treatment (*P* = 0.414). These results were in line with other clinical trials showing that hematological AEs increase with the bevacizumab treatment [17–20, 52, 53], and these toxicities are manageable.

There were several limitations with our study. First, it was a single-arm phase II clinical trial with limited statistical power. Second, recruited patients were rather heterogeneous in terms of prior chemotherapy regimens, with other likely weaknesses in patient’s characteristics inherent to a phase II design. Notwithstanding, it is unlikely that any of these limitations would substantially alter the qualitative nature of our conclusions.

In summary, our study was the first to evaluate bevacizumab-mFOLFOX6 regimen in heavily pretreated patients with HER2/neu-negative MBC, who were in a situation with very limited additional treatment options. For patients who were in good performance status at the study start, bevacizumab-mFOLFOX6 regimen showed high efficacy and good safety profile with promising objective responses rates, PFS and OS. For all these reasons, a robust controlled phase III trial based on large number of patients is guaranteed to confirm our results of the bevacizumab-mFOLFOX6 combination.

## Supporting Information

S1 CONSORT Checklist (DOC)

S1 Protocol Trial protocol approved by ethics committee, English version. (PDF)

S2 Protocol Trial protocol approved by ethics committee, Chinese version. (PDF)

S1 Table Clinical variables in univariate analysis. (DOCX)

S2 Table Clinical variables in multivariable analysis. (DOCX)

S3 Table Prior studies of chemotherapy regimens as second- or beyond line for MBC. (DOCX)

S4 Table Adverse events of present trial compared with those of our historical mFOLFOX6 trial. (DOCX)

S5 Table AEs of present trial in breast cancer compared with AEs in colorectal cancer. (DOCX)
