# Supplementary material for: Bevacizumab in Combination with Modified FOLFOX6 in Heavily Pretreated Patients with HER2/Neu-Negative Metastatic Breast Cancer: A Phase II Clinical Trial
Source: PLoS One. 2015 Jul 17;10(7):e0133133. doi: 10.1371/journal.pone.0133133 (PMC4506015; doi:10.1371/journal.pone.0133133)
Supplement: S1 Protocol — (PDF) [file pone.0133133.s002.pdf]

Bevacizumab in combination with modified FOLFOX6 in heavily pretreated patients with HER2/neu-negative metastatic breast cancer: a phase II clinical trial.

Department of Medical Oncology, Fudan University Shanghai Cancer Center

Study Chairman Xi-Chun Hu, MD

Coordinators Biyun Wang, Si Sun

1. BACKGROUND AND THEORETICAL FOUNDATION

1.1 Background

Breast cancer is one of the most malignant cancers in women globally, with 1.3 million new cases and 500,000 death each year. The incidence of breast cancer rank first in the developed countries, such as Western Europe and North America. Recently, the incident of breast cancer increased significantly in China, especially in some big cities. Due to the raised awareness and extensive medical screening, the rate of early diagnosis of breast cancer is increasing. However, there remain a percentage of patients who will eventually relapse and have distant metastasis. Metastatic breast cancer (MBC) is essentially an incurable disease with poor prognosis, with a median overall survival (OS) of 24-36 months and 5-year survival rate of 15-25%.

1.2 Treatment of metastatic breast cancer

The treatment is palliative for MBC. The main purpose is to prolong overall survival, reduce disease-related symptoms and improve the quality of life. Endocrine therapy is a favorable choice for hormone receptor-positive patients with a moderate activity and low toxicity. For patients with negative hormone receptor or endocrine-resistance, cytotoxic chemotherapy is the standard treatment. Taxanes and anthracyclines are the most effective agents for breast cancer with well-known benefits and toxicity profiles both in adjuvant and in first-line metastatic settings. Their efficacy in the first-line chemotherapy is established by the results of numerous clinical trials and retrospective studies. For the patients who do not respond or relapse early after the administration of taxane or anthracycline regimens, the agents recommended by the National Comprehensive Cancer Network (NCCN) guideline include vinorelbine, gemcitabine, and capecitabine, although no specific sequence of therapies has been advocated. However, the objective rate plummets after lines of chemotherapy. Therefore, for patients who have been heavily pretreated with the agents above but still in relatively good clinical condition, exploration of new combinations and schedules of drugs with proven efficacy are clearly needed.

1.3 Bevacizumab (Avastin) and treatment of metastatic breast cancer

The earliest study evaluating the safety and efficacy of bevacizumab in patients with previously treated metastatic breast cancer is a phase I/II trial. It enrolled 75 patients

treated with escalating doses of bevacizumab ranging from 3 mg/kg to 20 mg/kg every other week. The overall response rate was 9.3% and the median duration of confirmed response was 5.5 months. Three prior phase III trials (E2100, AVADO, RIBBON-1) have showed a good safety profile and a consistent improvement of progression free survival (PFS) and overall objective rate (ORR) in stage IV MBC patients treated with combination of bevacizumab and various chemotherapy agents as first-line treatment.

1.4 FOLFOX regimens and treatment of metastatic breast cancer

Oxaliplatin is the third generation of platinum after cisplatin and carboplatin. It has a synergistic effect with fluorouracil (5-FU). As a 5-FU sensitizer, leucovorin (CF) combined with 5-FU increase its anti-cancer activity. FOLFOX regimens composed of oxaliplatin, 5-FU and CF are widely used in gastrointestinal cancer. The previous phase II studies showed ORR of 27 to 34% and time to progression (TTP) of 4.8 to 5.3 months for these combinations in patients previously treated with anthracycline- or taxane-based chemotherapy, mostly in the second or third line on an every-3-week schedule. Our previously published study of modified FOLFOX6 for metastatic breast cancer showed an ORR of 18.3% and the median PFS of 3 months. Although our efficacy data were lower than data of other studies, patients enrolled in our trial were extremely heavily pretreated (58.1% ≥ 3 lines of chemotherapy) unlike those in the aforementioned studies. Therefore, the study demonstrated that the combination of oxaliplatin plus 5-FU/CF was a well-tolerated salvage regimen with moderate activity in patients with heavily pretreated MBC.

1.5 Bevacizumab (Avastin) combined with FOLFOX regimen and treatment of metastatic breast cancer

Bevacizumab combined with FOLFOX regimen has been widely used in the treatment of metastatic colorectal cancer. ECOG3200 showed that the addition of bevacizumab to oxaliplatin, 5-FU, and CF improved ORR, PFS and OS for metastatic colorectal cancer patients previously treated with a fluoropyrimidine and irinotecan. N016966 trial also showed that bevacizumab combined with FOLFOX/XELOX regimens as first line therapy improved PFS for metastatic colorectal cancer patients. With regard to safety, patients treated with combination experienced increased rates of hypertension,

proteinuria and bleeding events. However, they are mild and manageable.

In summary, bevacizumab and FOLFOX regimens are effective for metastatic breast cancer, the combined therapy has been proven with good safety and tolerability. The present study was to determine the efficacy and safety of bevacizumab-modified FOLFOX6 regimen in heavily pretreated patients with human epidermal growth factor receptor2-negative MBC.

2． STUDY OBJECTIVES

2.1 Primary Objectives

PFS (Progression Free Survival)：PFS was defined as the time from enrollment to the first documented disease progression or death from any cause.

2.2 Secondary Objectives

1. Objective response rate (ORR)：ORR was defined as the percentage of patients who achieved complete response (CR) and partial response (PR) by Response Evaluation Criteria in Solid Tumors (RECIST) version 1.1 criteria.
2. Clinical benefit rate (CBR): CBR was defined as the percentage of patients who achieved CR, PR and SD ≥24 weeks by RECIST version 1.1 criteria.
3. Overall survival (OS): OS was defined as the time from enrollment to the date of death from any cause.
4. Adverse events (AEs): AEs were evaluated, graded and recorded according to National Cancer Institute Common Terminology Criteria for Adverse Events (CTCAE), version 4.0.

3 PATIENT SELECTION

3.1 Inclusion Criteria

1. Provision of written informed consent prior to any study specific procedures
2. At least one measurable disease according to the RECIST criteria, version 1.1.
3. Histological proven HER-2 negative metastatic breast cancer and our hospital pathology consultation with written documents.
4. Females with age ≥18 years old
5. Enrolled patients had to have at least 2 prior chemotherapy regimens for metastatic disease, and were to be pretreated with anthracyclines and taxanes (including the

neoadjuvant chemotherapy, adjuvant chemotherapy or the chemotherapy for the metastatic settings). The researchers determine the appropriate patients for the trial.

6. Patients were required to complete all prior chemotherapy or radiotherapy at least 3 weeks before study entry or complete prior endocrine therapy at least 2 weeks before study entry.
7. ECOG performance status ≤ 2.
8. Life expectancy greater than 12 weeks.

3.2 Exclusion Criteria

1. Prior use of oxaliplatin or continuous infusion of 5-FU for metastatic breast cancer.
2. Prior use of xeloda with failure for metastatic breast cancer.
3. The cumulative doses of doxorubicin and epirubicin exceeded 360 mg/m2 and 720 mg/m2, respectively
4. Any toxicities remain greater than CTCAE grade I after previous anticancer therapy (including radiation therapy), expect alopecia and hematologic toxicities.
5. Use of other clinical trial medication or participating in other clinical trial currently or within the last 30 days.
5. History of major surgery (including open biopsy) within the last 28 days or minor surgery within the last 24 hours.
6. Absolute neutrophil count (ANC) $<1.5\times10^9$/L, platelet (PLT)$<80\times10^9$/L, hemoglobin (Hb) <8 g/dL.
7. Inadequate hepatic function:
   a) Serum bilirubin >1.5×upper limit of normal (UNL).
   b) Without liver metastasis, aspartate aminotransferase/ serum glutamic oxalacetic transaminase (AST/SGOT) or serum alanine transaminase/ serum glutamic pyruvic transaminase (ALT/SGPT) >2.5×UNL. With liver metastasis, AST/SGOT and ALT/SGPT>5×UNL.
   c) Without liver metastasis, alkaline phosphatase (ALP) >2.5×UNL. With liver metastasis, ALP>5×UNL. With bone metastasis, ALP>10×UNL.
8. Inadequate renal function:
   a) Serum creatinine >1.5×UNL.

b) Creatinine clearance rate <50 ml/min calculated according to Cockcroft-Gault formula.

9. Urinary protein score of more than 2+. If it is more than 2+ at baseline, it should be confirmed by 24-hour urine protein test. Patients with 24-hour urine protein of less than 1g can be enrolled.
10. Patients, who do not receive anticoagulation treatment, with an international normalized ratio (INR) of more than 1.5 or activated partial thromboplastin time (APTT) of more than 1.5×ULN at last 7 days.
11. Aspirin (>325mg/day) or clopidogrel (>75mg/day) intake for a long term.
12. Use of antiviral drugs such as sorivudine or its analogs with related chemical structure such as brivudine.
13. Use of corticosteroids (the dose of methylprednisolone or the equivalent dose of methylprednisolone ≥10mg/day) for a long term, except inhaled corticosteroids.
14. Evidence of spinal cord compression or brain metastases. If suspected, computed tomography (CT) or magnetic resonance imaging (MRI) scan should be taken within the last 4 weeks of study entry.
15. History of another malignancy within the last five years except carcinoma in-situ of uterine cervix and squamous cell carcinoma of skin after adequate therapy, or cured basal cell carcinoma of skin.
16. Pregnancy or breast feeding.
17. Uncontrolled seizures, history of central nervous system disorders or mental disorders.
18. History or evidence of inherited bleeding diathesis or coagulopathy with the risk of bleeding.
19. Uncontrolled hypertension (systolic blood pressure >140 mmHg and/or diastolic blood pressure >90 mmHg) or clinically significant or active cardiovascular and cerebrovascular diseases.
20. History of gastrointestinal fistula, grade IV intestinal obstruction, gastrointestinal perforation or abdominal abscess within the last 6 month.
21. Serious non-bleeding wound, peptic ulcer or bone fracture
22. Patients with active infection or other serious underlying medical conditions.

23. History of unpredictable severe adverse effect of fluorouracil. Prior dihypopyrimidine dehydrogenase deficiency.
24. Hypersensitivity to Chinese hamster ovary cell products or other recombinant human or humanlised antibodies
25. Evidence that may interfere with study treatment plan, impact patient compliance or increase treatment-related complications, such as mental illness, abnormalities of physical or laboratory examination.

4. SAMPLE SIZE

The pilot mFOLFOX6 regimen without bevacizumab showed a median PFS of 3 month. The planned sample size of 48 patients treated with the new bevacizumab-mFOLFOX6 regimen would allow detecting an increase of 2 months in the median PFS, with a 5% significance level at 90% power, 18 months enrollment and 6 month follow-up, based on two-sided test and 5% patient dropout rate.

5. STUDY PLAN

5.1 Treatment

Bevacizumab + modified FOLFOX6 (Avastin + mFOLFOX6)：

modified FOLFOX6

Oxaliplatin, 85 mg/m^2, intravenous drip over 2 hours, d1;
Leucovorin, CF, 400 mg/m^2, intravenous drip over 2 hours before 5-FU, d1;
5-FU, 400 mg/m^2, intravenous drip, d1, following 5-FU 2400 mg/m^2 intravenous drip over 46 hours, d1-2;
Repeat every 14 days, giving a maximum of 12 cycles.

Bevacizumab

Choose one of the two application programs below:

1）Bevacizumab (Avastin), 5mg/kg, d1, every 14 days, giving a maximum of 12 cycles.

2）Bevacizumab (Avastin), 7.5mg/kg, d1, every 21 days, giving a maximum of 8 cycles.

5.2 CONCOMITANT MEDICATIONS

1．Dexamethasone can be used to prevent vomiting. Other antiemetics are used according to local practice.

2. Granulocyte colony-stimulating factor (G-CSF) can be used for neutropenia, but not as a preventive medication.

3. Bisphosphonates for bone metastases confirmed by radiological evidence or ECT at baseline are allowed. Use of bisphosphonates after clinical trial entry with no bone metastases evidence at baseline is considered as disease progression.

4. Use of other cytotoxic drugs, clinical study drugs, active or passive immunotherapy and hormone therapy for breast cancer is not allowed. If radiotherapy is needed during the medication, it is considered as disease progression. Withdraw is required before radiotherapy start.

5. Other concomitant medications are determined by the principal investigators.

6. ASSESSMENT

6.1 Baseline assessment

Try to arrange all the checks and assessments close to the medication time.

Two weeks prior to the initial treatment:

History-taking of breast cancer, including age, menopausal status, fertility, date of first diagnosis, hormone receptor status, HER-2 status, date of relapse, previous treatment, metastatic sites at baseline, past and concomitant disease and medications.

Physical examination;

ECOG performance status;

Laboratory assessment and accessory examination: blood routine test (including Hb, red blood cells, white blood cells and PLT), urine routine test, liver function (total bilirubin, ALT, AST, ALP, albumin/globulin, lactic dehydrogenase), renal function (blood urea nitrogen, serum creatinine), blood glucose, serum electrolytes (K^+, Na^+, Cl^-), hemostasis (prothrombin time, APTT, INR), pregnancy test in pre-menopausal women, fertile women and women of menopause within the last 12 months, tumor markers (CEA, CA153, CA125), electrocardiogram and echocardiogram;

Scores of QOL;

Four weeks prior to the initial treatment:

Radiographic assessment: CT or MRI for disease evaluation should be performed within 4 weeks prior to the treatment, including head CT or MRI if indicated. If radionuclide is uptake in ECT, X-ray should be performed to rule out bone metastases. For patients receiving bisphosphonate therapy at baseline, ECT should be performed.

6.2 Treatment Assessment

6.2.1 One week prior to every cycle of mFOLFOX6 treatment

Physical examination;

Laboratory assessment and accessory examination: blood routine test, urine routine test, liver function (total bilirubin, ALT, AST, ALP, albumin/globulin, lactic dehydrogenase), renal function (blood urea nitrogen, serum creatinine), blood glucose, serum electrolytes (K+, Na+, Cl-), hemostasis (prothrombin time, APTT, INR), and electrocardiogram; left ventricular ejection fraction (LVEF) should be performed if clinically indicated. Patients, who have heart failure with symptoms, should repeat LVEF assessment every 2 weeks till the symptoms has been relived or stabilized.

6.2.2 Every four cycles of mFOLFOX6 treatment

Radiographic assessment: CT or MRI; echocardiogram;

Scores of QOL;

6.3 Interview at the end of study

Record physical examination, ECOG performance status, laboratory and radiographic assessment that is consistent with baseline; Record adverse events, concomitant medications, the date and reason of discontinuation and withdraw. If death occurs, record the reason.

Scores of QOL;

6.4 Exploratory biomarker research

1. Blood Sample for angiogenic biomarkers

The samples were collected (4ml for anticoagulant tube and 7ml for procoagulant tube respectively) two weeks prior to treatment or at the end of study for preparation of

plasma samples and serum samples to measure the angiogenesis biomarkers.

2. Tumor Sample

Tumor samples in the form of a paraffin embedded tissue (PET) block are collected for retrospective analysis of biomarkers.

6.5 Follow-up

Follow up all the patients enrolled and record the date of disease progression.

6.6 Duration of follow-up

The date of treatment starts till patients dead.

6.7 Frequency of follow-up

Every 3 months.

7 DOSE MODIFICATION AND TREATMENT DELAY

7.1 Bevacizumab

1. The dose of bevacizumab is calculated by baseline body weight and cannot be modified unless patient's body weight changes ±10% from baseline.
2. If toxicities occur, please refer to the drug manual of bevacizumab for guidance.
3. If severe toxicities (grade III/IV) of bevacizumab occur, such as hypertension, proteinuria, thrombosis/embolism, hemorrhage, congestive heart failure or wound healing complications and any other relevant toxicities related, bevacizumab may be temporarily or permanently discontinued.
4. Grade IV neutropenia and thrombocytopenia (whether related to the treatment or not) will lead to tendency of bleeding, so bevacizumab may be temporarily discontinued.
5. Once the following adverse events occur, bevacizumab should be permanently terminated:

   Brain reversible posterior leukoencephalopathy syndrome (RPLS)

   Grade III/IV bleeding/hemorrhage events

   Grade III/IV venous thrombosis

   Any grade of arterial thrombosis

Grade IV hypertension (hypertensive crisis)

Grade III proteinuria (nephrotic syndrome)

Grade III/IV left ventricular dysfunction (CHF)

Any grade gastrointestinal perforation

Any grade tracheoesophageal fistula

Any grade lung-gastrointestinal fistula

Any grade hypersensitivity/allergic reaction related with bevacizumab

7. Even if bevacizumab is terminated, chemotherapy can be continued.
8. Appropriate treatment of bevacizumab related adverse events

(a) Hypertension

The blood pressure must be closely monitored. Patients should rest ≥5 minutes before blood pressure measurement. If the initial systolic blood pressure ≥140mmHg and/or diastolic blood pressure ≥90mmHg, the measurement must be repeated to verify the results. If hypertension occurs, bevacizumab should be managed as described in Table 1.

(b) Proteinuria

Each time before bevacizumab treatment, evaluate 24 hours urine collection or urine proteinuria by urine test paper. Proteinuria should be treated according to Table 2

(c) Hemorrhage

If grade III/IV bleeding/ hemorrhage occurs, bevacizumab should be permanently terminated. If bleeding occurs under the application of full-dose anticoagulation therapy, bevacizumab should be permanently terminated and patients should be treated in accordance with guidelines of research center. Standard treatment includes protamine, antagonist of vitamin K, vitamin K-dependent factors or implantation of vena caval filter according to the severity of bleeding, thromboembolic events and affected organs.

7.2 mFOLFOX6 regimens

1. Dose modification of leucovorin is not allowed. The drug is administered before 5-FU treatment. If 5-FU delay, so as leucovorin.

2. Treatment should be interrupted for toxicities of any grade II or above (except alopecia and anemia) until it recovers to grade I or less.
3. For grade II anemia, chemotherapy is allowed. If there is grade III/IV anemia, treatment should be interrupted until toxicities recover to grade II or less.
4. If grade IV hematologic toxicity or febrile neutropenia occur, the dose of oxaliplatin and 5-FU should be reduced by 25%.
5. If any grade III/IV non-hematologic toxicities (except neurotoxicity) occur, the dose of the related drug should be reduced by 25% and the other drugs remain the same.
6. If grade III neurologic toxicity occurs, the dose of oxaliplatin in the next cycle should be reduced by 25%. If grade IV neurologic toxicity occurs, oxaliplatin should be terminated.

7.3 Treatment Discontinuation

Patients will be discontinued from the study treatment and follow-up until death under the following circumstances:

1. Maximum allowed treatment cycle (6 cycles) is completed;
2. Patients who need further dose modification after 2 times;
3. Disease progression during the study
4. Unacceptable/ intolerable adverse events
5. Pregnancy
6. Protocol violations that render the patient unsuitable for further treatment;
7. Any other reason deemed appropriate by the investigator.

7.4 Study Withdrawal

Patients will be withdrawn from the study under the following circumstances:

1. Patient’s withdrawal of informed consent
2. Intercurrent illness, which would significantly affect assessments of clinical status
3. Any other reason deemed appropriate by the investigator
4. Other non-protocol systemic cancer therapy or prohibited medication is initiated.

8. EVALUATION AND CALCULATION OF VARIABLES

8.1 Primary objective

Progression free survival (PFS)

8.2 Secondary objectives

Objective response rate (ORR)

Clinical benefit rate (CBR)

Overall survival (OS)

8.3 Safety

The incidence of adverse events

The incidence of serious adverse events

Laboratory parameters

Vital Signs

Total dose, actual dose/planned dose

The time and frequency of reduction

9. POPULATION ANALYSES

9.1 ITT (Intent-to-Treat) population

All the patients enrolled.

9.2 PP (Per-Protocol）population

Population excluding the patients of ITT population who do not receive study treatment or do not meet the inclusion criteria and exclusion criteria.

9.3 Safety assessment population

Patients who received at least one dose of study treatment.

9.4 Prognostic factor analysis

The prognostic factors impacting PFS and survival include:

ECOG performance status (0,1 vs 2)

Metastatic sites at baseline (visceral metastases yes vs. no)

Hormone receptor status (ER or PR (+) vs ER and PR (-))

Number of metastatic sites at baseline (<3 vs ≥ 3)

10. SUPPLY OF STUDY DRUGS

Study drugs are available in market.

11. TREATMENT AFTER STUDY

The patients, who did not have disease progress at the end of study, will receive maintenance therapy or stop medication and be followed up, according to guidelines. Bevacizumab is optional for the maintenance treatment. For hormone receptor positive patients, endocrine therapy is applicable for maintenance therapy. The treatment after study should be recorded in the case report form (CRF).

Table 1

CTCAE 4.0	Hypertension	Treatment
Grade I	Prehypertension (systolic BP 120-139mmHg or diastolic BP 80-89mmHg)	Giving bevacizumab;
Grade II	Stage I hypertension (systolic BP 140-159mmHg or diastolic BP 90-99mmHg); medical intervention indicated; recurrent or persistent (≥24 hours); symptomatic increase > 20mmHg (diastolic) or to >140/90mmHg if previously within normal limits; monotherapy indicated;	Stop bevacizumab and give anti-hypertensive therapy until hypertension back to grade I;
Grade III	Stage II hypertension (systolic BP ≥160mmHg or diastolic BP ≥ 100mmHg); medical intervention indicated; more than one drug or more intensive therapy than previously used indicated;	Stop bevacizumab and give anti-hypertensive therapy until hypertension back to grade I; If it fails to control the high blood pressure, bevacizumab should be permanently terminated.
Grade IV	Life-threatening consequences; urgent intervention indicated;	Bevacizumab should be permanently terminated.

Table 2

CTCAE 4.0	Treatment
Grade I	Giving bevacizumab;
Grade II	For urinary protein score of more than 2+, give bevacizumab and confirm the 24-hour urine protein next cycle before bevacizumab treatment;<br>For urinary protein score of more than 3+, confirm the 24-hour urine protein before bevacizumab treatment;<br>If proteinuria ≥2g/24 hour, stop bevacizumab until proteinuria <2g/24 hours.
Grade III	Bevacizumab should be permanently terminated.

12. REFERENCES

1. Parkin DM, et al. Global cancer statistics 2002. CA Cancer J Clin. 2005;55:74–108.

2. Kamby C, et al: metastatic pattern in recurrent breast cancer. Special reference to intrathoracic recurrences. Cancer 1988; 62: 2226–33.

3. Jemal,A. et al. Cancer statistics 2007. CA Cancer J Clin 57, 43-66.

4. Cardoso,F. et al. Second and subsequent lines of chemotherapy for metastatic breast cancer: what did we learn in the last two decades? Ann Oncol 2002; 13: 197-207.

2. National Comprehensive Cancer Network. Clinical Practice Guidelines in Oncology: Breast cancer v.2.2011. http://www.nccn.org/professionals/physician_gls/PDF/breast.pdf. (2011).

3. Garufi C, et al. Single-agent oxaliplatin in pretreated advanced breast cancer patients: a phase II study. Ann Oncol 2001; 12: 179–82.

4. Zelek L, et al. Phase II study of oxaliplatin and fluorouracil in taxane- and

anthracycline-pretreated breast cancer patients. J Clin Oncol 2002; 20: 2551–8.

5. Pectasides D, et al. Oxaliplatin plus high-dose leucovorin and 5-fluorouracil in pretreated advanced breast cancer: a phase II study. Ann Oncol 2003; 14: 537–42.

6. Sun S, et al. Phase II study of oxaliplatin plus leucovorin and 5-fluorouracil in heavily pretreated metastatic breast cancer patients. Med Oncol. Published online: 06 Feb 2012

7. De Gramont A, et al. Leucovorin and fluorouracil with or without oxaliplatin as first-line treatment in advanced colorectal cancer. J Clin Oncol 2000; 18: 2938–47.

8. Martı´n M, et al. Gemcitabine plus vinorelbine versus vinorelbine monotherapy in patients with metastatic breast cancer previously treated with anthracyclines and taxanes: final results of the phase III Spanish breast cancer research group (GEICAM) trial. Lancet Oncol 2007; 8: 219–25.

9. Leonardi V, et al. Oxaliplatin combined with 5-fluorouracil and folinic acid in pretreated advanced breast cancer patients: preliminary reports. Ann N Y Acad Sci 2002; 963: 91–7.

10. Extra JM, et al. Pharmacokinetics and safety profile of oxaliplatin. Semin Oncol 1998; 25: 13–22.

11. Cobleigh M.A, et al. A phase I/II dose-escalation trial of bevacizumab in previously treated metastatic breast cancer. Semin Oncol 2003; 30: 117-24.

12. Miller K, et al: Paclitaxel plus bevacizumab versus paclitaxel alone for metastatic breast cancer. N Engl J Med 2007; 357: 2666-2676

13. Miles DW, et al. Phase III study of bevacizumab plus docetaxel compared with placebo plus docetaxel for the first-line treatment of human epidermal growth factor receptor 2-negative metastatic breast cancer. J Clin Oncol 2010; 28: 3239-47

14. Robert NJ, et al. RIBBON-1: randomized, double-blind, placebo-controlled, phase III trial of chemotherapy with or without bevacizumab for first-line treatment of human epidermal growth factor receptor 2-negative, locally recurrent or metastatic breast cancer. J Clin Oncol. 2011; 29:1252-60

15. Giantonio BJ, et al. Bevacizumab in combination with oxaliplatin, fluorouracil, and leucovorin (FOLFOX4) for previously treated metastatic colorectal cancer: Results from the Eastern Cooperative Oncology Group Study E3200. J Clin Oncol 2007; 25:1539-1544

16. Saltz LB，et al. Bevacizumab in Combination With Oxaliplatin-Based Chemotherapy As First-Line Therapy in Metastatic Colorectal Cancer: A Randomized Phase III Study. J Clin Oncol 2008; 26:2013-2019

B-3（1.0 版）

复旦大学附属肿瘤医院医学伦理委员会
伦理委员会签到及申明

第 111 次伦理会议

会议时间：2012.7.2　11：30　　　　会议地点：2 号楼 5 楼第五会议室

姓名 Name	性别/sex	职务(伦理委员会) /Position in EC	专业 /Profession Background	签名/signature	日期/date
吴炅 Wu Jiong	男/male	主任委员/Chair	外科 Surgery	——	请假
杜祥 Du Xiang	男/male	副主任委员/Vice Chair	病理 Pathology	——	请假
洪小南 Hong Xiaonan	女/female	委员/member	内科 Medical Oncology	洪小南	2012.7.2
胡超苏 Hu Chaosu	男/male	委员/member	放疗 Radiation Oncology	[illegible]	2012.7.2
师英强 Shi Yingqiang	男/male	委员/member	外科 Surgery	[illegible]	2012.7.2
王华英 Wang Huaying	女/female	委员/member	妇科 Gynecology	[illegible]	2012.7.2
陆箴琦 Lu Zhenqi	女/female	委员/member	护理 Nursing	陆箴琦	2012.7.2
印季良 Yin Jiliang	女/female	委员/member	内科 Medical Oncology	——	请假
郭晔 Guo Ye	男/male	委员/member	内科 Medical Oncology	[illegible]	2012.7.2
孟志强 Meng zhiqiang	男/male	委员/member	中西医结合 Integrative Oncology	[illegible]	2012.7.2
彭卫军 Peng Weijun	男/male	委员/member	放射诊断 Diagnostic Radiology	[illegible]	2012.7.2
朱骥 Zhu Ji	男/male	委员/member	放疗、统计 Radiation Oncology & Statistics	朱骥	2012.7.2
翟青 Zhai Qin	女/female	委员/member	药学 Pharmacy	翟青	2012.7.2
杨绍刚 Yang Shaogang	男/male	委员/member	律师 Lawyer	[illegible]	2012.7.2
刁月琴 Diao Yueqin	女/female	委员/member	社区工作 Community Cadres	刁月琴	2012.7.2
陆琴 Lu Qin	女/female	委员秘书 /member secretary	科研管理 Science &Research Administration	陆琴	2012.7.2
张玮静 Zhang Weijing	女/female	秘书/secretary	科研管理 Science & Research Administration	张玮静	2012.7.2

伦理委员会申明：

- 复旦大学附属肿瘤医院伦理委员会组成及工作程序遵循 ICH-GCP 及中国相关法律法规
- 伦理委员会委员已签署同意信守机密或商业专有秘密，并同意该信息只以伦理审查为目的，不用于其他目的或公开给第三方。提供审查用的书面机密资料不能被复制或保留。所有标准操作规程文件、机密信息、摘记等及其副本的所有权均归伦理委员会。

B-1（1.0版）

复旦大学附属肿瘤医院医学伦理委员会
批准函

伦理编号：1204109-2-1207A

审查日期	2012.7.2
审查会议地点	2号楼5楼第五会议室
研究项目名称	贝伐单抗联合改良FOLFOX6方案挽救治疗HER-2阴性的复发转移性乳腺癌的II期临床试验
审查文件	相关资料（每单项必须填写，提交资料标记为 ✓，未提交资料的标记为 ╳，如无版本号标记为 —）<br>☒ 国家食品药品监督管理局批件，批件文号：_<br>☑ 方案，版本号：1.2版(2012.5.30)<br>☑ 项目，编号：2012-20-579<br>☒ 药品生产许可证及检测报告/医疗器械注册证及检验报告<br>☑ 知情同意书样本，版本号：1.2版(2012.5.30)<br>☒ 研究者履历、临床研究经历<br>☒ 其他（请说明）：______
研究科室	肿瘤内科
主要研究者	胡夕春
申办者	研究者发起
伦理审查方式	☑会议审查　　□快速审查
投票结果	应到人数：15人　　实到人数：12人<br>回避委员：无
审查意见	同意方案及知情同意书修正，新版方案、知情同意书从即日起实施。<br>主任委员签字：[illegible]<br>复旦大学附属肿瘤医院医学伦理委员会（盖章）<br>日期：2012年7月2日

注意：（请仔细阅读）

1. 该研究进行过程中将接受伦理委员会的持续审查，审查频度为研究批准之日起：/
（伦理委员会有权根据实际进展情况改变持续审查频度）
2. 本批件将在各中心机构及其伦理委员会备案。
3. 已批准项目须遵循本伦理委员会批准的方案执行，须符合SFDA/GCP和《赫尔辛基宣言》的原则。
4. 暂停/提前终止临床研究，请及时通知伦理委员会。
5. 发生严重不良事件及影响研究风险受益比的非预期事件，须及时报告本伦理委员会。
6. 对已批准的临床研究方案、知情同意书等材料的任何修改及主要研究者更换等，须及时通知本伦理委员会重新审查，获得批准后执行。
7. 发现违反方案情况须及时报告伦理委员会。
8. 根据伦理委员会对持续审查频度的意见，无论试验开始与否，请在持续审查日到期前1个月提出持续审查的申请。
9. 完成临床研究，须提交结题报告供伦理委员会审查。

附件：伦理委员会签到及保密协议、伦理委员会组成人员名单
