# Supplementary material for: Bevacizumab in Combination with Modified FOLFOX6 in Heavily Pretreated Patients with HER2/Neu-Negative Metastatic Breast Cancer: A Phase II Clinical Trial
Source: PLoS One. 2015 Jul 17;10(7):e0133133. doi: 10.1371/journal.pone.0133133 (PMC4506015; doi:10.1371/journal.pone.0133133)
Supplement: S2 Protocol — (PDF) [file pone.0133133.s003.pdf]

贝伐单抗联合改良FOLFOX6方案挽救治疗HER-2阴性的复发转移性乳腺癌的II期临床试验

复旦大学附属肿瘤医院肿瘤内科

主要负责人：胡夕春

协调研究者：王碧芸，孙思

1. 背景及理由

1.1 背景

乳腺癌是女性最常见的恶性肿瘤之一，全世界每年约有130万新发病例，50万死亡病例。在西欧、北美等发达国家，乳腺癌发病率占女性恶性肿瘤首位。我国近年来发病率增长明显，在一些大城市已经位列女性肿瘤发病首位。由于认识的提高和筛查的广泛开展，乳腺癌的早期诊断率正在不断提高。然而，仍有一定比例的患者最终将复发并出现远处转移。转移性乳腺癌（MBC）患者的预后较差，且被认为是不可治愈的，中位总生存期在24-36个月左右，5 年生存率很低，只有15-25%。

1.2 转移性乳腺癌的治疗现状

对转移性乳腺癌的患者而言，治疗在本质上为姑息性。主要目的是延长生存期、减少疾病相关症状以及提高患者的生活质量。在常规的临床治疗中，对激素受体阳性的肿瘤，内分泌治疗由于其疗效较好、便于使用以及毒性极低而成为通常的选择。对激素受体阴性或激素受体阳性但内分泌耐药后患者,细胞毒性化疗是标准的治疗。蒽环类和紫杉类被认为是乳腺癌最有效的治疗药物之一，其单药或联合治疗方案通常用于初始治疗。对于紫杉类或蒽环类耐药的患者，可选择的化疗药物包括卡培他滨、长春瑞滨和吉西他滨等，但无标准治疗方案。在应用序贯多线化疗的情况下有效率不断下降，但许多患者仍有良好的体力状况，迫切需要得到进一步治疗，因此有必要研究新的药物及优化药物组合。

1.3 贝伐单抗（Avastin）与转移性乳腺癌的治疗

最早对贝伐单抗进行评价的临床研究是一项Ⅰ/Ⅱ期剂量递增临床试验,包括75例曾经接受过治疗的转移性乳腺癌患者。患者分别接受3mg/kg、10 mg/kg或20 mg/kg贝伐单抗（q2w）单药治疗，总有效率为9.3%，中位疗效维持时间为5.5个月。在转移性乳腺癌中已报道了3个贝伐单抗联合化疗的III期随机临床研究（E2100、AVADO、RIBBON-1）与单用化疗组相比，贝伐单抗联合化疗组其能显著延长患者的无进展生存期（PFS），并提高客观缓解率（ORR），且患者耐受性良好，安全可靠。

1.4 FOLFOX方案与转移性乳腺癌的治疗

奥沙利铂是继顺铂和卡铂之后第三代铂类抗癌药。奥沙利铂与氟尿嘧啶(5－FU)有协同效应，亚叶酸钙（CF）作为 5-Fu 增效剂，与 5-Fu 合用可增加其抗癌活性。奥沙利铂、5－FU 和 CF 组成的 FOLFOX 方案广泛应用于胃肠道肿瘤，有良好的治疗效果。在转移性乳腺癌的治疗中，有多项 II 期临床研究证实对于紫杉类或蒽环类耐药的患者，奥沙利铂联合 5FU 为基础的方案具有中度活性，ORR 为 27% 到 34%，中位至疾病进展时间（TTP）为 4.8 至 5.3 个月，其不良反应低，可作为多线治疗失败后选择。本中心的一项 II 期单中心临床研究应用 FOLFOX 方案治疗多药耐药的转移性乳腺癌患者，ORR 为 18.3%，PFS 为 3 个月，略低于文献报道。其原因可能在于本组患者病期晚，且大多数患者接受了三线以上治疗，考虑到其良好的安全性及耐受性，应不失为一个良好的解救治疗方案。

1.5 贝伐单抗（Avastin）联合 FOLFOX 方案与转移性乳腺癌的治疗

贝伐珠单抗联合 FOLFOX 方案已广泛应用于转移性结直肠癌治疗中。 ECOG3200 研究证明了在既往接受伊立替康和氟尿嘧啶治疗的晚期结直肠癌患者，贝伐单抗联合 FOLFOX 与单独接受 FOLFOX 相比,能明显提高 ORR,PFS 及总生存期(OS)。NO16966 研究也表明贝伐单抗联合 FOLFOX/XELOX 与单独化疗相比,能明显提高晚期结直肠癌一线治疗的 PFS。 安全性方面，尽管联合治疗组增加了高血压、蛋白尿、出血的风险，但大多数情况下是轻微而易于控制。

综上所述，贝伐单抗和 FOLFOX 方案对转移性乳腺癌均有效，其联合治疗疗效已得到肯定，且表现出良好的安全性和耐受性。本研究将应用贝伐单抗联合 FOLFOX6 方案治疗一线治疗失败的 HER-2 阴性转移性乳腺癌，其研究结果值得期待。

2. 研究目的

2.1 主要目的

无进展生存期 （PFS）：定义为自首次研究药物给药起至首次证实疾病进展或死亡的时间。

2.2 次要目的

1. 总有效率（ORR）：定义为根据实体瘤疗效评价标准1.1（RESCIST1.1）评价后

获得完全缓解（CR）或部分缓解（PR）的患者的百分比。

2. 临床获益率（CBR）：定义为RESCIST1.1评价后获得稳定（SD）不少于24周、客观有效（CR或PR）的患者的百分比。
3. 总生存期（OS）：定义为维持治疗期首次研究用药给药起至任何原因导致死亡的时间。
4. 评价毒性：根据美国国立癌症研究所不良反应常用术语标准 (NCI-CTCAE) 4.0 版进行分级

3 病例选择

3.1 入选标准

1. 在筛选前先获得病人的知情同意书。
2. 按RECIST 1.1标准，至少有一个可测量病灶。
3. 具有经组织学确认且经我院病理科会诊，有书面文件证明的HER-2 阴性的转移性乳腺癌患者。
4. 年龄≥18 岁的女性患者。
5. 患者经至少二线化疗后失败，且既往治疗（包括新辅助，辅助及复发转移后治疗）中使用过蒽环类及紫杉类药物。研究者判断适合运用本研究方案的治疗者。
6. 距先前的化疗或放疗至少3周，既往内分泌治疗结束至少2周。
7. ECOG 评分 0, 1或2。
8. 预计生存＞12 周。

3.2 排除标准

1. 既往针对转移性乳腺癌曾接受奥沙利铂或持续5FU静脉滴注治疗。
2. 既往针对转移性乳腺癌曾接受希罗达治疗且希罗达治疗无效。
3. 既往治疗含有以蒽环类药物为基础的患者，治疗的最大累积剂量多柔比星超过360mg/m2，表柔比星超过 720mg/m2。
4. 既往抗癌治疗（包括放射治疗）留下任何未缓解＞CTCAE 1 级的毒性，脱发和血液学毒性除外。
5. 目前或最近（距离本次临床研究首次给药 30 天以内）应用其他临床研究药物治疗或参加其他临床研究。
5. 距首次临床研究治疗前 28 天内进行较大的外科手术操作（包括开放性活检），或首次临床研究治疗前 24 小时内接受较小的外科手术。

6. 骨髓功能不良：绝对中性粒细胞计数（ANC）＜1.5 × 109/L，或血小板计数＜80×109/L 或血红蛋白＜8 g/dL。
7. 肝功能不全，定义为：
   a) 血清（总）胆红素＞1.5×正常值上限（ULN）
   b) 血清谷草转氨酶（AST/SGOT）或谷丙转氨酶（ALT/SGPT）＞2.5 ×ULN（肝转移患者＞5×ULN）
   c) 碱性磷酸酶水平在基线时＞2.5 ×ULN（肝转移患者＞5×ULN，或骨转移患者＞10×ULN）
8. 肾功能不全，定义为：
   a) 血清肌酐＞1.5 ×ULN
   b) 肌酐清除率＜50 ml/min（根据 Cockcroft-Gault 公式计算）
9. 尿试纸检测尿蛋白＞2+。基线评价时尿试纸分析尿蛋白 2+的患者应再进行 24 小时尿液收集，若证实 24 小时尿蛋白＜1g 可入组。
10. 未接受抗凝治疗的患者国际标化比（INR）＞1.5 或活化的部分凝血活酶时间（APTT）在首次临床研究给药前 7 天以内＞1.5 ×ULN。
11. 每日阿司匹林（＞325mg/天）或氯吡格雷（＞75mg/天）长期治疗。
12. 需要同时应用抗病毒药物索立夫定或相关化学结构类似物（如溴夫定）。
13. 长期每日应用皮质类固醇（甲基强的松龙或等效剂量≥10mg/天），但吸入性糖皮质激素除外。
14. 有脊髓压迫或脑转移的证据。如果怀疑这些部位存在转移，必须在首次临床研究给药前 4 周内进行脑部的 CT 或 MRI 扫描。
15. 最近 5 年以内伴有其他恶性肿瘤，但经过充分治疗的宫颈原位癌或皮肤鳞癌、或已基本控制的皮肤基底细胞癌除外。
16. 妊娠或哺乳期女性。
17. 具有未控制的癫痫发作、中枢神经系统功能紊乱病史，或精神障碍患者。
18. 证实有遗传性出血素质或有出血风险的凝血疾病史的患者。
19. 不能控制的高血压（收缩压＞140 mmHg 和/或舒张压＞90 mmHg）或具有临床意义的（即活动性的）心、脑血管疾病。
20. 距首次临床研究治疗 6 个月以内有腹腔瘘、4 度肠梗阻、胃肠道穿孔或腹腔内脓肿的病史。
21. 严重的非愈合性创伤、消化性溃疡或骨折。
22. 在入组期间发生需要抗生素静脉注射治疗的严重活动性感染。

23. 既往曾发生对氟尿嘧啶治疗无法预测的严重反应。
24. 对中国仓鼠卵巢细胞产物或其他重组人或人源化抗体过敏。
25. 具有其他可能干扰治疗计划、影响患者依从性或使患者存在治疗相关并发症高风险的医学条件（如精神疾病、体格检查或实验室检查异常）的证据。

4. 病例数

根据本研究主要终点指标 PFS 估计样本量，根据文献报道，FOLFOX 方案治疗转移性乳腺癌的 PFS 约为 3 个月，假设联合治疗组延长 2 个月达到 5 个月。按双侧检验，I 类错误 5%，把握度 90%，病例入组需 18 个月，受试者全部入组后继续随访 6 个月，考虑 5%的失访，本试验共需 48 例患者。

5. 试验方案

5.1 方案

贝伐单抗 + 改良 FOLFOX6 （Avastin + mFOLFOX6）：

改良 FOLFOX6

奥沙利铂（Oxaliplatin）85 mg/m^2，静滴 2 小时，第 1 天；

亚叶酸钙（Leucovorin，CF）400 mg/m^2，在氟尿嘧啶（5-FU）前 2 小时静滴，第 1 天；

氟尿嘧啶（5-FU）400 mg/m^2，静脉注射，第 1 天，然后再用 2400 mg/m^2，46 小时持续静滴，第 1～2 天；

每 14 天一次，最多给予 12 个周期。

贝伐单抗

本研究可以从两种贝伐单抗应用方案中选择

1）贝伐单抗 (Avastin) 每公斤体重 5 毫克（5mg/kg），第 1 天，每 14 天一次，最多给予 12 个周期。

2）贝伐单抗 (Avastin) 每公斤体重 7.5 毫克（7.5mg/kg），第 1 天，每 21 天一次，最多给予 8 个周期。

5.2 合并用药

1. 地塞米松可用于预防呕吐。止吐药物按当地惯例。

2. 粒细胞集落刺激因子 G-CSF 可治疗有症状的粒缺，但不能作为预防性用药。
3. 使用双膦酸盐的病人需在基线时有影像学或 ECT 证实骨转移。如基线时无骨转移证据而在治疗当中开始运用双膦酸盐，则被视为疾病进展。
4. 不允许应用其他细胞毒药物、其他研究药物、乳腺癌的主动和被动免疫或激素治疗。研究当中如需要放疗则被视为病情进展，放疗开始前需出组。
5. 其他合并用药由主要研究者决定。

6. 观察指标和检查时间

6.1 入组前的检查

所有检查尽量安排与治疗开始时间越接近越好。

在首次治疗前 2 周内：

病史采集；包括年龄，月经状态，生育能力，乳腺癌病史包括首次诊断、激素受体状态，HER-2 状态、诊断和复发的日期，乳腺癌的既往治疗，基线时肿瘤的转移部位。既往和伴随的疾病及药物治疗。

全身体格检查；

ECOG 评分；

实验室检查及辅助检查：血常规（包括血红蛋白、红细胞、白细胞计数及分类、血小板计数）、尿常规、肝功能（TBL、ALT、AST、AKP、A/G、LDH）、肾功能（BUN、Cr、）、血糖、血电解质（K+、Na+、Cl-）、凝血功能（PT、APTT、INR））血或尿妊娠试验（绝经前妇女，有生育能力的女性，和绝经后尚未达到 12 个月无月经的女性），肿瘤标记物（CEA,CA153,CA125），心电图和心脏超声；

生活质量评分。

在首次治疗前 4 周内：

影像学检查：主要采用 CT 或 MRI 检查。如有临床指征行骨扫描和脑的 CT/MRI。如 ECT 有核素浓聚，应行 X 线检查以除外骨转移。基线时曾接受双膦酸盐治疗的患者，应行骨扫描。

6.2 研究期间的检查

6.2.1 每个 mFOLFOX6 治疗周期开始前 1 周内

全身体格检查；

实验室检查及辅助检查：血常规、尿常规、肝功能（TBL、ALT、AST、AKP、A/G 、LDH）、肾功能（BUN、Cr）、凝血功能（PT、APTT、INR），血糖、血电解质（K+、Na+、

Cl-)，心电图；左室射血分数仅在有临床指征的情况下需要重复检查。而如果出现症状性心力衰竭，则每 2 周重复检查左室射血分数直至病情缓解或稳定。

6.2.2 每 4 个 mFOLFOX6 周期治疗结束时

影像学检查：CT 或 MRI 检查；心脏超声检查。

生活质量评分。

6.3 试验结束时访视

体格检查、ECOG 评分，各项实验室检查及辅助检查（同筛选检查）；影像学检查：在研究结束时确认，影像学检查应与入组时的检查方法相同。记录不良事件、伴随用药。对中止参加或退出研究的受试者，记录治疗中断原因、中止日期、退出日期及原因，如发生死亡应注明死亡原因。

生活质量评分。

6.4 生物标记物测量

1. 用于血管生成生物标记物测定的血标本

   将在首次治疗前 2 周内和试验结束时采集静脉血（分别为抗凝管 4ml 和促凝管 7ml）制备血浆标本和血清标本，测定血管生成的生物标记物。

2. 用于保存肿瘤的标本

   在患者同意后收集用石蜡包埋保存的肿瘤标本，用于回顾性生物标记物的分析。

6.5 随访

该项研究对全部受试者进行随访，记录疾病进展的时间。

6.6 随访起止时间

治疗开始，直至患者死亡。

6.7 随访频度

每 3 个月随访一次。

7 剂量调整和延迟治疗

7.1 贝伐单抗

1. 将使用基线体重计算所需的剂量，且预期不能进行剂量调整，除非患者的体重

较基线变化± 10%。

2. 如果出现毒副反应，请参考贝伐单抗药物说明书以获得指导。
3. 如下所述，如果出现高血压、蛋白尿、血栓形成/栓塞、出血、充血性心力衰竭或伤口愈合并发症以及其他任何与贝伐单抗有关的严重毒副反应（3 或 4 级），则贝伐单抗可能暂时或永久性停止使用。
4. 如果出现 4 级中性粒细胞减少或 4 级血小板减少症（不论是否与治疗有关），由于这些情况是增加出血倾向的诱发因素，则贝伐单抗应暂停使用。
5. 此外，在发生过以下事件的患者中应该永久性终止贝伐单抗的治疗：

   可逆性大脑后部白质脑病综合征（RPLS）

   3/4 级失血/出血事件

   3/4 级 静脉血栓

   任何程度的动脉血栓形成

   4 级高血压（高血压危象）

   3 级蛋白尿（肾病综合征）

   3/4 级左心功能不全（CHF）

   任何程度的胃肠道穿孔

   任何程度的气管食管瘘

   任何程度的肺-胃肠瘘

   任何程度的与贝伐单抗有关的超敏/变态反应

7. 即使终止贝伐单抗治疗，化疗仍可以继续应用。
8. 贝伐单抗相关事件的适当处理方式如下所述

   (a) 高血压

   在研究期间必须密切监测患者高血压发展与恶化的情况。血压应在患者休息体位≥5 分钟后进行测定。如果初始血压读数收缩压≥140mmHg 和/或舒张压≥90mmHg，必须重复测定以验证结果。如果发生高血压，贝伐单抗的治疗应该如表 1 所描述的进行管理。

   (b) 蛋白尿

   在每一次贝伐单抗治疗开始前，除非已进行 24 小时尿液收集评价，否则应通过尿试纸评价蛋白尿的情况。应根据表 2 处理蛋白尿。

   (c) 出血

   如果患者在研究治疗期间发生 3 或 4 级任何类型的出血，则必须永久性终止贝伐单抗的治疗。如果患者在应用全剂量抗凝药物治疗时发生出血并发

症，必须永久性终止贝伐珠单抗的治疗并按照研究中心的指南进行治疗。标准治疗包括应用鱼精蛋白或维生素 K 拮抗、输注维生素 K 依赖因子或根据出血的严重程度、血栓事件及受影响的器官考虑插入腔静脉滤器。

7.2 mFOLFOX6 方案

1. 亚叶酸钙不因毒性而调整，药物在 5-FU 用药前给药，如果 5-FU 推迟，亚叶酸钙也推迟应用。
2. 对于任何 CTCAE 2 级或以上毒性（脱发、贫血除外）应中断治疗，待毒性恢复至 1 级以内再开始治疗。
3. 对于 CTCAE 2 级贫血，可继续化疗。若出现 3/4 级贫血，则待毒性恢复到 2 级以内再继续治疗。
4. 出现 4 级血液学毒性或者中性粒细胞减少性发热，下一疗程奥沙利铂和 5FU 剂量降低 25%
5. 出现 3/4 级非血液学毒性（神经毒性除外），如果研究者认为毒性仅与一个药物有关，则不需要调整其他药物的剂量，下一疗程相关药物减量 25%。
6. 出现 3 级神经毒性，下一疗程奥沙利铂剂量降低 25%，出现 4 级神经毒性，则停用奥沙利铂。

7.3 终止治疗

在下述情况下，患者需终止治疗并随访至死亡：

1. 已完成最长允许的治疗；
2. 经两次调整剂量仍不能耐受的患者；
3. 给药时间延迟 2 周以上的患者；
4. 试验中病情进展（PD）；
5. 试验中发生不能耐受的不良反应；
6. 妊娠；
7. 由于违反研究方案所致的患者不适合接受进一步治疗（由研究者决定）；
8. 其他研究者认为有必要终止治疗的情况。

7.4 退出研究

在下述情况下，患者将终止治疗和退出研究：

1. 患者撤回知情同意；
2. 发生严重影响临床评价的并发疾病；
3. 任何研究者认为有必要退出该项研究的情况；
4. 接受其它非本研究的全身治疗或使用本研究禁用的药物。

8. 观察指标

8.1 主要指标

无进展生存期 （PFS）

8.2 次要指标

总有效率（ORR）

临床获益率（CBR）

总生存期（OS）

8.3 安全性指标:

不良事件发生率

严重不良事件发生率

实验室指标

生命体征

总剂量，实际剂量/计划剂量

减量的时间和发生频率

9. 分析人群

9.1 ITT（Intent-to-Treat ）人群:

所有入组的病人.

9.2 PP （Per-Protocol）人群

排除了没有接受任何研究治疗或违反入排标准的 ITT 病人。

9.3 安全性人群

所有接受了至少一次研究治疗的人群。

9.4 预后因子分析

影响 PFS 和生存期的预后因子包括：

ECOG 评分（0，1 vs 2）

基线时的转移部位（有内脏转移 vs 无内脏转移）

激素受体状态（ER 或 PR（+） vs 两者均（-））

基线时转移部位数目（＜3 个 vs ≥3 个）

10. 试验药品的提供

试验药品均为市售药品。

11. 后续治疗

治疗结束后未进展的患者按照诊疗常规，可选择维持治疗，也可停药观察。对于维持治疗的选择，可以选择贝伐单抗进行维持治疗，对于激素受体阳性的患者也可选择内分泌药物维持治疗。后续治疗方案记录在 CRF 表中。

表　　1

CTCAE 4.0	高血压模式	治　　疗
1 级	高血压前期（收缩压 120－139mmHg 或舒张压 80－89mmHg）	给予贝伐单抗治疗
2 级	I 期高血压，血压收缩压在 140-159 mmHg 或舒张压 90-99 mmHg；血压升高需要医学干预；复发性或持续性（＞24 小时）升高；有症状性升高，升高幅度＞20 mmHg（舒张压）或血压＞140/90 但以前血压在正常范围内；需要单一治疗手段	暂停贝伐单抗，给予抗高血压治疗，待毒性恢复到 1 级后继续贝伐单抗治疗
3 级	II 级高血压（收缩压≥160mmHg 或舒张压≥100mmHg）；血压升高需要医学干预；需要一种以上的药物治疗或强度比以前大的治疗	暂停贝伐单抗，抗高血压治疗后如恢复 1 级可继续贝伐单抗治疗。如高血压未能控制，则永久性终止贝伐单抗治疗。
4 级	危及生命的后果；需要紧急医学干预。	永久性终止贝伐单抗

表 2

CTCAE 4.0	措 施
1 级	给予贝伐单抗治疗
2 级	对于尿蛋白 2+，给予贝伐单抗治疗，并在下次给予贝伐单抗之前收集 24 小时尿液评价<br>对于对于尿蛋白 3+，进行收集 24 小时尿液收集评价后方可给予贝伐单抗治疗。<br>若蛋白尿≥2g/24 小时，暂停贝伐单抗治疗，并在蛋白尿<2g/24 小时时恢复治疗。
3 级	永久性终止贝伐单抗

12. 参考文献

1. Parkin DM, et al. Global cancer statistics 2002. CA Cancer J Clin. 2005;55:74–108.

2. Kamby C, et al: metastatic pattern in recurrent breast cancer. Special reference to intrathoracic recurrences. Cancer 1988; 62: 2226–33.

3. Jemal,A. et al. Cancer statistics 2007. CA Cancer J Clin 57, 43-66.

4. Cardoso,F. et al. Second and subsequent lines of chemotherapy for metastatic breast cancer: what did we learn in the last two decades? Ann Oncol 2002; 13: 197-207.

2. National Comprehensive Cancer Network. Clinical Practice Guidelines in Oncology: Breast cancer v.2.2011. http://www.nccn.org/professionals/physician_gls/PDF/breast.pdf. (2011).

3. Garufi C, et al. Single-agent oxaliplatin in pretreated advanced breast cancer patients: a phase II study. Ann Oncol 2001; 12: 179–82.

4. Zelek L, et al. Phase II study of oxaliplatin and fluorouracil in taxane- and anthracycline-pretreated breast cancer patients. J Clin Oncol 2002; 20: 2551–8.

5. Pectasides D, et al. Oxaliplatin plus high-dose leucovorin and 5-fluorouracil in pretreated advanced breast cancer: a phase II study. Ann Oncol 2003; 14: 537–42.

6. Sun S, et al. Phase II study of oxaliplatin plus leucovorin and 5-fluorouracil in heavily pretreated metastatic breast cancer patients. Med Oncol. Published online: 06 Feb 2012

7. De Gramont A, et al. Leucovorin and fluorouracil with or without oxaliplatin as first-line treatment in advanced colorectal cancer. J Clin Oncol 2000; 18: 2938–47.

8. Martı´n M, et al. Gemcitabine plus vinorelbine versus vinorelbine monotherapy in patients with

metastatic breast cancer previously treated with anthracyclines and taxanes: final results of the phase III Spanish breast cancer research group (GEICAM) trial. Lancet Oncol 2007; 8: 219–25.

9. Leonardi V, et al. Oxaliplatin combined with 5-fluorouracil and folinic acid in pretreated advanced breast cancer patients: preliminary reports. Ann N Y Acad Sci 2002; 963: 91–7.

10. Extra JM, et al. Pharmacokinetics and safety profile of oxaliplatin. Semin Oncol 1998; 25: 13–22.

11. Cobleigh M.A, et al. A phase I/II dose-escalation trial of bevacizumab in previously treated metastatic breast cancer. Semin Oncol 2003; 30: 117-24.

12. Miller K, et al: Paclitaxel plus bevacizumab versus paclitaxel alone for metastatic breast cancer. N Engl J Med 2007; 357: 2666-2676

13. Miles DW, et al. Phase III study of bevacizumab plus docetaxel compared with placebo plus docetaxel for the first-line treatment of human epidermal growth factor receptor 2-negative metastatic breast cancer. J Clin Oncol 2010; 28: 3239-47

14. Robert NJ, et al. RIBBON-1: randomized, double-blind, placebo-controlled, phase III trial of chemotherapy with or without bevacizumab for first-line treatment of human epidermal growth factor receptor 2-negative, locally recurrent or metastatic breast cancer. J Clin Oncol. 2011; 29:1252-60

15. Giantonio BJ, et al. Bevacizumab in combination with oxaliplatin, fluorouracil, and leucovorin (FOLFOX4) for previously treated metastatic colorectal cancer: Results from the Eastern Cooperative Oncology Group Study E3200. J Clin Oncol 2007; 25:1539-1544

16. Saltz LB，et al. Bevacizumab in Combination With Oxaliplatin-Based Chemotherapy As First-Line Therapy in Metastatic Colorectal Cancer: A Randomized Phase III Study. J Clin Oncol 2008; 26:2013-2019

B-3（1.0版）

复旦大学附属肿瘤医院医学伦理委员会
伦理委员会签到及申明

第 111 次伦理会议

会议时间：2012.7.2　11：30　　　　会议地点：2号楼5楼第五会议室

姓名<br>Name	性别/sex	职务(伦理委员会)<br>/Position in EC	专业<br>/Profession<br>Background	签名/signature	日期/date
吴炅<br>Wu Jiong	男/male	主任委员/Chair	外科<br>Surgery	——	请假
杜祥<br>Du Xiang	男/male	副主任委员/Vice Chair	病理<br>Pathology	——	请假
洪小南<br>Hong Xiaonan	女/female	委员/member	内科<br>Medical Oncology	洪小南	2012.7.2
胡超苏<br>Hu Chaosu	男/male	委员/member	放疗<br>Radiation Oncology	[illegible]	2012.7.2
师英强<br>Shi Yingqiang	男/male	委员/member	外科<br>Surgery	[illegible]	2012.7.2
王华英<br>Wang Huaying	女/female	委员/member	妇科<br>Gynecology	[illegible]	2012.7.2
陆箴琦<br>Lu Zhenqi	女/female	委员/member	护理<br>Nursing	陆箴琦	2012.7.2
印季良<br>Yin Jiliang	女/female	委员/member	内科<br>Medical Oncology	——	请假
郭晔<br>Guo Ye	男/male	委员/member	内科<br>Medical Oncology	[illegible]	2012.7.2
孟志强<br>Meng zhiqiang	男/male	委员/member	中西医结合<br>Integrative Oncology	[illegible]	2012.7.2
彭卫军<br>Peng Weijun	男/male	委员/member	放射诊断<br>Diagnostic Radiology	[illegible]	2012.7.2
朱骥<br>Zhu Ji	男/male	委员/member	放疗、统计<br>Radiation Oncology &<br>Statistics	朱骥	2012.7.2
翟青<br>Zhai Qin	女/female	委员/member	药学<br>Pharmacy	翟青	2012.7.2
杨绍刚<br>Yang Shaogang	男/male	委员/member	律师<br>Lawyer	[illegible]	2012.7.2
刁月琴<br>Diao Yueqin	女/female	委员/member	社区工作<br>Community Cadres	刁月琴	2012.7.2
陆琴<br>Lu Qin	女/female	委员秘书<br>/member secretary	科研管理<br>Science &Research<br>Administration	陆琴	2012.7.2
张玮静<br>Zhang Weijing	女/female	秘书/secretary	科研管理<br>Science & Research<br>Administration	张玮静	2012.7.2

伦理委员会申明：

- 复旦大学附属肿瘤医院伦理委员会组成及工作程序遵循ICH-GCP及中国相关法律法规
- 伦理委员会委员已签署同意信守机密或商业专有秘密，并同意该信息只以伦理审查为目的，不用于其他目的或公开给第三方。提供审查用的书面机密资料不能被复制或保留。所有标准操作规程文件、机密信息、摘记等及其副本的所有权均归伦理委员会。

B-1（1.0版）

复旦大学附属肿瘤医院医学伦理委员会
批准函

伦理编号：1204109-2-1207A

审查日期	2012.7.2
审查会议地点	2号楼5楼第五会议室
研究项目名称	贝伐单抗联合改良FOLFOX6方案挽救治疗HER-2阴性的复发转移性乳腺癌的II期临床试验
审查文件	相关资料（每单项必须填写，提交资料标记为 ✓，未提交资料的标记为 ╳，如无版本号标记为 —）<br>☒ 国家食品药品监督管理局批件，批件文号：_<br>☑ 方案，版本号：1.2版(2012.5.30)<br>☑ 项目，编号：2012-20-579<br>☒ 药品生产许可证及检测报告/医疗器械注册证及检验报告<br>☑ 知情同意书样本，版本号：1.2版(2012.5.30)<br>☒ 研究者履历、临床研究经历<br>☒ 其他（请说明）：______
研究科室	肿瘤内科
主要研究者	胡夕春
申办者	研究者发起
伦理审查方式	☑会议审查　□快速审查
投票结果	应到人数：15人　实到人数：12人<br>回避委员：无
审查意见	同意方案及知情同意书修正，新版方案、知情同意书从即日起实施。<br>主任委员签字：[signature]<br>复旦大学附属肿瘤医院医学伦理委员会（盖章）<br>日期：2012年7月2日

注意：（请仔细阅读）

1. 该研究进行过程中将接受伦理委员会的持续审查，审查频度为研究批准之日起：/
（伦理委员会有权根据实际进展情况改变持续审查频度）
2. 本批件将在各中心机构及其伦理委员会备案。
3. 已批准项目须遵循本伦理委员会批准的方案执行，须符合SFDA/GCP和《赫尔辛基宣言》的原则。
4. 暂停/提前终止临床研究，请及时通知伦理委员会。
5. 发生严重不良事件及影响研究风险受益比的非预期事件，须及时报告本伦理委员会。
6. 对已批准的临床研究方案、知情同意书等材料的任何修改及主要研究者更换等，须及时通知本伦理委员会重新审查，获得批准后执行。
7. 发现违反方案情况须及时报告伦理委员会。
8. 根据伦理委员会对持续审查频度的意见，无论试验开始与否，请在持续审查日到期前1个月提出持续审查的申请。
9. 完成临床研究，须提交结题报告供伦理委员会审查。

附件：伦理委员会签到及保密协议、伦理委员会组成人员名单
