# Supplementary material for: Bevacizumab in Combination with Modified FOLFOX6 in Heavily Pretreated Patients with HER2/Neu-Negative Metastatic Breast Cancer: A Phase II Clinical Trial
Source: PLoS One. 2015 Jul 17;10(7):e0133133. doi: 10.1371/journal.pone.0133133 (PMC4506015; doi:10.1371/journal.pone.0133133)
Supplement: S1 Table — (DOCX) [file pone.0133133.s004.docx]

**S1 Table Clinical variables in univariate analysis.**

| **Variables** | **Number of patients (%)** | **median PFS (95% CI)** | ***P* value^*^** | **median OS (95% CI)** | ***P* value^**^** |
| --- | --- | --- | --- | --- | --- |
| Age |  |  | 0.310 |  | 0.681 |
| ≥ 60 years | 14 (20.3) | 5.6 (3.4-7.8) |  | 12.8 (7.5-18.0) |  |
| < 60 years | 55 (79.7) | 6.9 (5.3-8.6) |  | 10.4 (8.9-11.9) |  |
| ECOG performance status |  |  | 0.333 |  | 0.250 |
| < 2 | 63 (91.3) | 6.8 (5.1-8.4) |  | 11.2 (8.6-13.8) |  |
| 2 | 6 (8.7) | 3.3 (0.0-10.8) |  | 6.7 (0.0-15.1) |  |
| Disease-free interval |  |  | 0.102 |  | 0.439 |
| > 12 months | 47 (74.6) | 7.1 (5.6-8.7) |  | 10.3 (8.8-11.9) |  |
| ≤ 12 months | 16 (25.4) | 2.9 (0.0-7.0) |  | 8.4 (5.0-11.8) |  |
| Molecular subtype |  |  | 0.061 |  | 0.311 |
| TNBC | 32 (46.4) | 4.3 (1.2-7.4) |  | 10.3 (8.6-12.1) |  |
| Non-TNBC | 37 (53.2) | 7.1 (5.6-8.7) |  | 11.2 (8.1-14.4) |  |
| Menstruation status |  |  | 0.108 |  | 0.711 |
| Post-menopausal | 43 (62.3) | 5.6 (3.7-7.5) |  | 10.3 (7.4-13.3) |  |
| Pre-menopausal | 26 (37.7) | 7.2 (5.9-8.4) |  | 11.3 (7.5-15.0) |  |
| Visceral metastasis |  |  | 0.783 |  | 0.908 |
| Yes | 58 (84.1) | 5.6 (3.2-8.0) |  | 10.5 (7.6-13.4) |  |
| No | 11 (15.9) | 7.8 (6.5-9.0) |  | 11.3 (5.3-17.4) |  |
| Number of metastatic sites |  |  | 0.805 |  | 0.142 |
| < 3 | 25 (36.2) | 6.5 (4.5-8.4) |  | 13.4 (9.0-17.7) |  |
| ≥ 3 | 44 (63.8) | 6.8 (4.2-9.3) |  | 10.0 (7.4-12.7) |  |
| Objective response status |  |  | **0.000** |  | **0.005** |
| ORR | 31 (44.9) | 8.2 (7.4-9.0) |  | 14.1 (11.6-16.6) |  |
| Non-ORR | 38 (55.1) | 4.0 (3.4-4.6) |  | 8.4 (5.5-11.3) |  |
| Lines |  |  | 0.103 |  | 0.233 |
| 2 | 21 (30.4) | 6.9 (5.0-8.9) |  | 11.3 (6.2-16.4) |  |
| ≥ 3 | 48 (69.6) | 6.0 (3.4-8.6) |  | 10.3 (8.9-11.8) |  |
| Pretreated with anthracyclines |  |  | 0.255 |  | 0.264 |
| Yes | 65 (94.2) | 6.8 (5.0-8.5) |  | 10.5 (8.0-13.0) |  |
| No | 4 (5.8) | 4.0 (1.8-6.3) |  | 6.4 (0.0-13.2) |  |
| Pretreated with taxanes |  |  | **0.044** |  | 0.925 |
| Yes | 68 (98.6) | 6.8 (5.2-8.3) |  | 10.5 (9.2-11.8) |  |
| No | 1 (1.4) | 2.6 (NA-NA) |  | 13.4 (NA-NA) |  |
| Hypertension |  |  | 0.132 |  | 0.052 |
| Yes | 8 (11.6) | 9.9 (0.0-20.1) |  | 15.4 (9.3-21.5) |  |
| No | 61 (88.4) | 6.4 (5.0-7.9) |  | 10.3 (8.8-11.9) |  |
| Proteinuria |  |  | 0.158 |  | 0.163 |
| Yes | 11 (15.9) | 4.9 (1.8-7.9) |  | 10.0 (6.6-13.5) |  |
| No | 58 (84.1) | 6.8 (5.6-8.0) |  | 11.3 (8.7-13.9) |  |

Abbreviations: PFS, progression free survival; ECOG, eastern cooperative oncology group; TNBC, triple-negative breast cancer; ORR, objective response rate; NA, not applicable. ^*^ *P* value for PFS; ^**^ *P* value for OS.
