# Supplementary material for: Bevacizumab in Combination with Modified FOLFOX6 in Heavily Pretreated Patients with HER2/Neu-Negative Metastatic Breast Cancer: A Phase II Clinical Trial
Source: PLoS One. 2015 Jul 17;10(7):e0133133. doi: 10.1371/journal.pone.0133133 (PMC4506015; doi:10.1371/journal.pone.0133133)
Supplement: S2 Table — (DOCX) [file pone.0133133.s005.docx]

**S2 Table Clinical variables in multivariable analysis.**

| **Variables** | **HR (95% CI)^*^** | ***P* value^*^** | **HR (95% CI)^**^** | ***P* value^**^** |
| --- | --- | --- | --- | --- |
| Molecular subtype (Non-TNBC v.s. TNBC) | 0.484 (0.281-0.833) | **0.009** | - | - |
| Objective response status (ORR v.s. Non-ORR) | 0.268 (0.153-0.468) | **0.000** | 0.335 (0.185-0.606) | **0.000** |
| Number of metastatic sites | 1.210 (1.004-1.459) | **0.045** | 1.420 (1.156-1.743) | **0.001** |
| Age | - | **-** | 0.954 (0.926-0.983) | **0.002** |

Abbreviations: PFS, progression free survival; TNBC, triple-negative breast cancer; ORR, objective response rate. ^*^ Value for PFS; ^**^ Value for OS.
