# Supplementary material for: Bevacizumab in Combination with Modified FOLFOX6 in Heavily Pretreated Patients with HER2/Neu-Negative Metastatic Breast Cancer: A Phase II Clinical Trial
Source: PLoS One. 2015 Jul 17;10(7):e0133133. doi: 10.1371/journal.pone.0133133 (PMC4506015; doi:10.1371/journal.pone.0133133)
Supplement: S3 Table — (DOCX) [file pone.0133133.s006.docx]

**S3 Table Prior studies of chemotherapy regimens as second- or beyond line for MBC**

| **Study** | **Regimens** | **Line** | ***No.* of Patients** | **ORR (%)** | **median PFS (months)** | **median OS (months)** |
| --- | --- | --- | --- | --- | --- | --- |
| Li *et al* | Bevacizumab+mFOLFOX6 | 3, 2-7 | 69 | 50.0 | 6.8 | 10.5 |
| Sun *et al*[5] | mFOLFOX6 | 4, 2-7 | 62 | 18.3 | 3 | 10 |
| Aogi *et al* [37] | Eribulin | ≦2 | 25 | 36.0 | 3.7 | 14.6 |
|  |  | 3 | 34 | 14.7 | 4.0 | 11.3 |
|  |  | 4 | 21 | 14.3 | 1.9 | 7.3 |
| Cortes *et al* [38] | Eribulin | ≥2 | 269 | 14.1 | 2.6 | 10.4 |
| Seo *et al*[39] | Vinorelbine | 3, 2-5 | 26 | 20.8 | 3.7(median TTP) | 10.4 |
| Fumoleau *et al*[40] | Vinflunine | 3 | 56 | 14 | 2.6 | 11.4 |
| Modi *et al*[41] | Gemcitabine | 3, 2-5 | 22 | 17 | NA | 9.5 |
| Rha *et al* [42] | Gemcitabine | 2-3 | 38 | 20 | 4.5 | 11.0 |
| Smorenburg *et al* [43] | Gemcitabine | 3 | 23 | 0 | 1.9 (median TTP) | 7.8 |
| Rivera *et al* [44] | Paclitaxel | 3 | 35 | 20 | NA | NA |
|  |  | ≥4 | 33 | 18 | NA | NA |
| Abrams *et al* [45] | Paclitaxel | ≥3 | 172 | 23 | NA | NA |

Abbreviations: NA, not applicable; TTP, time to progression; MBC, metastatic breast cancer; ORR, objective response rate; PFS, progression free survival; OS, overall survival.
