# Supplementary material for: Bevacizumab in Combination with Modified FOLFOX6 in Heavily Pretreated Patients with HER2/Neu-Negative Metastatic Breast Cancer: A Phase II Clinical Trial
Source: PLoS One. 2015 Jul 17;10(7):e0133133. doi: 10.1371/journal.pone.0133133 (PMC4506015; doi:10.1371/journal.pone.0133133)
Supplement: S4 Table — (DOCX) [file pone.0133133.s007.docx]

**S4 Table Adverse events of present trial compared with those of our historical mFOLFOX6 trial**

| **Toxicities** | **Sun *et al* [5]** | | **Li *et al*** | |
| --- | --- | --- | --- | --- |
|  | **Grade 1-4, *N* (%)** | **Grade 3-4, *N* (%)** | **Grade 1-4, *N* (%)** | **Grade 3-4, *N* (%)** |
| Anemia | 14 (22.5) | 0 (0.0) | 34 (49.3) * | 3 (4.3) |
| Neutropenia | 35 (56.5) | 14 (22.6) | 64 (92.8) * | 53 (76.8) * |
| Thrombocytopenia | 25 (40.3) | 9 (14.5) | 37 (53.6) | 13 (18.8) |
| Nausea/vomiting | 13 (21.0) | 1 (1.6) | 37 (53.6) * | 0 (0.0) |
| Diarrhea | 8 (12.9) | 1 (1.6) | 18 (26.1) | 0 (0.0) |
| Fever | 7 (11.3) | 0 (0.0) | 8 (11.6) | 0 (0.0) |
| Neuropathy | 13 (21.0) | 3 (4.8) | 28 (40.6) * | 0 (0.0) |

Fisher’s exact tests, *<0.05
