# Supplementary material for: Bevacizumab in Combination with Modified FOLFOX6 in Heavily Pretreated Patients with HER2/Neu-Negative Metastatic Breast Cancer: A Phase II Clinical Trial
Source: PLoS One. 2015 Jul 17;10(7):e0133133. doi: 10.1371/journal.pone.0133133 (PMC4506015; doi:10.1371/journal.pone.0133133)
Supplement: S5 Table — (DOCX) [file pone.0133133.s008.docx]

**S5 Table AEs of present trial in breast cancer compared with those of in colorectal cancer**

| **Grade 3-4 AEs** | **Li *et al***  ***N* =69** | **Hochster *et al* [9]**  ***N* =71** | **Schmoll *et al* [49]**  ***N* =704** | **Allegra *et al* [50]**  ***N* =1326** | **Yamada *et al* [51]**  ***N* =249** |
| --- | --- | --- | --- | --- | --- |
| Anemia, *N* (%) | 3 (4.3) | 0 (0) | NA | NA | 6 (2) |
| Leukopenia, *N* (%) | 36 (52.2) | 5 (7)* | NA | NA | 21 (8)* |
| Thrombocytopenia, *N* (%) | 13 (18.8) | 2 (3)* | 18 (3)* | 19 (1.4)* | 2(1)* |
| Neutropenia, *N* (%) | 53 (76.8) | 35 (49)* | 166 (24)* | 390 (29.4)* | 84 (34)* |
| Febrile Neutropenia, *N* (%) | 2 (2.9) | 2 (3) | NA | 16 (1.2) | NA |
| Bleeding, *N* (%) | 0 (0.0) | 2 (3) | 10 (1.4) | 25 (1.9) | NA |
| Cardiac events, *N* (%) | 0 (0.0) | NA | NA | 20 (1.5) | NA |
| Proteinuria, *N* (%) | 0 (0.0) | NA | 6 (0.9) | 36 (2.7) | 0 (0) |
| Hypertension, *N* (%) | 3 (4.3) | 5 (7) | 29 (4) | 159 (12.0) | 14 (6) |
| Thromboembolism, *N* (%) | 0 (0.0) | 3 (4) | 59 (8.4) | 84 (6.3) | 4(2) |
| Gastrointestinal perforation, *N* (%) | 0 (0.0) | NA | 8 (1.1) | 4 (0.3) | 1 (<1) |
| Wound complications, *N* (%) | 1 (1.4) | NA | NA | 23 (1.7) | NA |
| Discontinuation due to AEs, *N* (%) | 7 (10.1) | 32 (45)* | 148 (21)* | NA | NA |

Abbreviation: AEs, adverse events; NA, not applicable.

Fisher’s exact tests, *<0.05
